# Blood-based protein biomarkers for the diagnosis of acute stroke: A discovery-based SWATH-MS proteomic approach

**DOI:** 10.3389/fneur.2022.989856

**Published:** 2022-09-27

**Authors:** Shubham Misra, Praveen Singh, Manabesh Nath, Divya Bhalla, Shantanu Sengupta, Amit Kumar, Awadh K. Pandit, Praveen Aggarwal, Achal K. Srivastava, Dheeraj Mohania, Kameshwar Prasad, Deepti Vibha

**Affiliations:** ^1^Department of Neurology, All India Institute of Medical Sciences, New Delhi, India; ^2^CSIR-Institute of Genomics and Integrative Biology, New Delhi, India; ^3^Department of Emergency Medicine, All India Institute of Medical Sciences, New Delhi, India; ^4^Dr. R.P. Centre, All India Institute of Medical Sciences, New Delhi, India; ^5^Department of Neurology, Rajendra Institute of Medical Sciences, Ranchi, Jharkhand, India

**Keywords:** stroke, ischemic stroke, intracerebral hemorrhage, proteomics, blood biomarkers, SWATH-MS

## Abstract

**Background and purposes:**

Recent developments in high-throughput proteomic approach have shown the potential to discover biomarkers for diagnosing acute stroke and to elucidate the pathomechanisms specific to different stroke subtypes. We aimed to determine blood-based protein biomarkers to diagnose total stroke (IS+ICH) from healthy controls, ischemic stroke (IS) from healthy controls, and intracerebral hemorrhage (ICH) from healthy control subjects within 24 h using a discovery-based SWATH-MS proteomic approach.

**Methods:**

In this discovery phase study, serum samples were collected within 24 h from acute stroke (IS & ICH) patients and healthy controls and were subjected to SWATH-MS-based untargeted proteomics. For protein identification, a high-pH fractionated peptide library for human serum proteins (obtained from SCIEX) comprising of 465 proteins was used. Significantly differentially expressed (SDE) proteins were selected using the following criteria: >1.5-fold change for upregulated, < 0.67 for downregulated, *p-*value < 0.05, and confirmed/tentative selection using Boruta random forest. Protein–protein interaction network analysis and the functional enrichment analysis were conducted using STRING 11 online tool, g:Profiler tool and Cytoscape 3.9.0 software. The statistical analyses were conducted in R version 3.6.2.

**Results:**

Our study included 40 stroke cases (20 IS, 20 ICH) within 24 h and 40 age-, sex-, hypertension-, and diabetes-matched healthy controls. We quantified 375 proteins between the stroke cases and control groups through SWATH-MS analysis. We observed 31 SDE proteins between total stroke and controls, 16 SDE proteins between IS and controls, and 41 SDE proteins between ICH and controls within 24 h. Four proteins [ceruloplasmin, alpha-1-antitrypsin (SERPINA1), von Willebrand factor (vWF), and coagulation factor XIII B chain (F13B)] commonly differentiated total stroke, IS, and ICH from healthy control subjects. The most common significant pathways in stroke cases involved complement and coagulation cascades, platelet degranulation, immune-related processes, acute phase response, lipid-related processes, and pathways related to extracellular space and matrix.

**Conclusion:**

Our discovery phase study identified potential protein biomarker candidates for the diagnosis of acute stroke and highlighted significant pathways associated with different stroke subtypes. These potential biomarker candidates warrant further validation in future studies with a large cohort of stroke patients to investigate their diagnostic performance.

## Introduction

Stroke is a medical emergency in which brain cells die rapidly post its onset. It is broadly classified based on its etiology into two types: (1) ischemic stroke (IS)—occlusion of the artery supplying oxygen-rich blood to the brain resulting in brain cell or tissue death within minutes; and (2) intracerebral hemorrhage (ICH)—rupturing of the blood vessel that bleeds into the surrounding brain leading to further brain damage (1). Despite the two stroke subtypes sharing a similar risk profile (2), they exhibit distinct molecular mechanisms in the acute phase (3–6). Thus, an efficient and rapid diagnosis of stroke is warranted within the first few hours of symptom onset for the effective treatment strategies to be implemented to prevent adverse outcomes. Due to the unavailability of neuroimaging facilities in most developing nations and time-sensitive nature of revascularization therapies, blood biomarkers are needed to aid clinical decision-making. Biomarkers detected in the blood may also help in elucidating the molecular mechanisms underlying the two stroke subtypes.

Recent developments in high-throughput proteomic approaches have shown the potential to discover biomarkers for diagnosing acute stroke and to elucidate the pathomechanisms specific to different stroke subtypes. The label-free approach using data-independent acquisition (DIA) method acquires superior peptide peaks compared to conventional proteomic data-dependent acquisition (DDA) methods and allows screening of a broad range of protein biomarkers with high reproducibility and efficiency.

Few studies in the past have utilized the high-throughput proteomic approaches for blood biomarker identification in stroke (7–11). However, these studies were conducted beyond the 24-h time window and failed to identify the expression pattern of proteins in the acute phase of stroke. Majority of these studies pooled their samples for proteomic analysis, which might lead to false-positive or false-negative results as pooled samples do not reflect the diseased/non-diseased state of a single person (10, 11). Therefore, our exploratory study aimed to determine blood-based protein biomarkers related to the pathogenesis of stroke in the acute phase of onset. Our goal was to provide a list of candidate protein markers that can diagnose and differentiate total stroke (IS + ICH) from healthy controls, IS from healthy controls, and ICH from healthy control subjects within 24 h of symptom onset using a discovery-based SWATH-MS proteomic approach without pooling any sample. We used an age-, sex-, and risk factor- (hypertension and diabetes) matched healthy control group, to identify biomarker expression pattern specific to stroke pathophysiology.

## Methods

The study was conducted at the Department of Neurology, All India Institute of Medical Sciences, New Delhi, India, from August 2016 to August 2021 in collaboration with Institute of Genomics and Integrative Biology (IGIB), New Delhi, India. Stroke patients aged 18 years and above, ischemic or hemorrhagic confirmed by neuroimaging and clinical diagnosis admitted within 24 h of symptom onset to the neurology wards and/or emergency department of AIIMS, New Delhi, were included in the study. All included patients had clinical signs consistent with the definition of stroke given by the Stroke Council of American Heart Association (AHA)/ American Stroke Association (ASA) (12). A control group comprising of age- (±2 years), sex-, hypertension-, and diabetes-matched individuals was taken from subjects in the general outpatient department (OPD) with no prior history of any neurological disorder and was evaluated by questionnaire for verifying stroke-free status (QVSFS) (13). A written informed consent was taken from all the subjects included in the study prior to collecting blood samples and clinical history.

### Sample size

The literature suggests a sample size of 10 to 30 to be adequate for conducting an exploratory/discovery phase study (14, 15). Therefore, based on the feasibility, budget, and time frame of the study, the sample size for the discovery phase was kept as 40 per group consisting of 40 stroke (20 IS and 20 ICH) and 40 control subjects.

### Blood sample collection

After the written informed consent was obtained, 5 ml of peripheral blood samples was taken in serum vacutainer tubes from 20 IS and 20 ICH patients admitted within 24-h onset of stroke. Blood samples were also taken from 40 healthy individuals who served as controls for the study. For serum collection, it was left standing at room temperature for 30 min until clotted. It then underwent centrifugation at 3,000 rpm for 10 min, after which the serum was separated into serum-containing vials. Five aliquots of each sample (100 μl) were prepared and stored at −80°C until further analysis.

### Sample preparation

Ten μl of serum samples was used for protein precipitation. To 90μl of 1X phosphate buffer saline (PBS), 10 μl serum was added and vortex mixed. Protein precipitation was performed using pre-chilled acetone. Briefly, to 100 μl protein extract, four times volume of pre-chilled acetone was added, vortex mixed, and centrifuged at 15,000 g for 10 min at 4°C. The supernatant was discarded, and the protein pellets were air-dried at room temperature and suspended in 0.1 M Tris-HCl with 8 M urea and pH 8.5. Protein quantitation was performed using the Bradford assay.

### Reduction, alkylation, and trypsin digestion

A total of 20 μg of protein from each sample were reduced with 25 mM of dithiothreitol (DTT) for 30 min at 60°C, followed by alkylation using 55 mM of iodoacetamide (IAA) at room temperature (in the dark) for 30 min. These samples were then subjected to trypsin digestion in an enzyme to substrate ratio of 1:10 (trypsin: protein) for 16–18 h at 37°C. Finally, the tryptic peptides were vacuum-dried in vacuum concentrator.

### Sequential window acquisition of all theoretical fragment ion spectra-mass spectrometry (SWATH-MS) data acquisition

Peptides from each sample were cleaned up using C18 ZipTip (Merck) using the manufacturer's protocol. SWATH-MS analysis (16) for the samples was performed on a quadrupole-TOF hybrid mass spectrometer (TripleTOF 6600, SCIEX) coupled to an Eksigent NanoLC-425 system. Optimized source parameters were used, and curtain gas and nebulizer gas were maintained at 25 psi and 30 psi, respectively. The ion spray voltage was set to 5.5 kV, and the temperature was set to 250°C. About 4 μg of peptides was loaded on a trap column (ChromXP C18CL 5 μm 120 Å, Eksigent, SCIEX), and online desalting was performed with a flow rate of 10 μl per min for 10 min. Next, the peptides were separated on a reverse-phase C18 analytical column (ChromXP C18, 3 μm 120 Å, Eksigent, SCIEX) in 57 min long gradient with a flow rate of 5 μl/min using water with 0.1% formic acid and acetonitrile with 0.1% formic acid.

SWATH method was created with 95 precursor isolation windows, defined based on precursor m/z frequencies in DDA run using the SWATH Variable Window Calculator (SCIEX), with a minimum window of 5 m/z. Data were acquired using Analyst TF 1.7.1 Software (SCIEX). Accumulation time was set to 250 msec for the MS scan (400–1,250 m/z) and 25 msec for the MS/MS scans (100–1,500 m/z). Rolling collision energies were applied for each window based on the m/z range of each SWATH and a charge 2+ ion, with a collision energy spread of five. The total cycle time was 3.37 s.

### Bioinformatic and statistical analyses

For identification of the proteins using SWATH analysis, a high-pH fractionated peptide library for human serum proteins (obtained from SCIEX) comprising of 465 proteins was used. SWATH peaks were extracted using this library in SWATH 2.0 microapp in PeakView 2.2 software (SCIEX), excluding shared peptides. SWATH run files were added, and retention time calibration was performed using peptides from abundant proteins. The peptide query parameters (PQPs) for peak extraction were as follows: maximum of 10 peptides per protein, five transitions per peptide, >95% peptide confidence threshold, and 1% peptide false discovery rate (FDR). XIC extraction window was set to 55 min with 75 ppm XIC Width. These PQPs were derived from the high-pH fractionated peptide library for peptide identification. All information was exported in the form of MarkerView (mrkw) files. In MarkerView 1.2.1 (SCIEX), data normalization was performed using total area sum normalization for internal correction and exported to excel. The data were log_2_ transformed to account for naturally skewed intensity values.

Batch correction for removing the non-biological experimental variations including the sample batches run at different timepoints was performed using the “ComBat” function inside the “sva” package (17) in R version 3.6.2. The principal component analysis (PCA) plots for the batch uncorrected and batch corrected data were plotted using the “prcomp” function inside the “factoextra” package (18) in R version 3.6.2. Significant differences between the means of the two groups were calculated using a *t*-test.

Significantly differentially expressed proteins were selected using two criteria: (i) *p-*value < 0.05 and ± 1.5-fold change (>1.5 for upregulated and < 0.67 for downregulated proteins) cutoffs wherein significantly upregulated/downregulated proteins were visualized using the volcano plot created in R version 3.6.2; or (ii) confirmed/tentative selection in the Boruta random forest feature selection method using the “Boruta” package (19) in R version 3.6.2.

The STRING 11 online tool (Search Tool for the Retrieval of Interacting Genes/Proteins 11) (20) was used to create the protein network of the significantly differentially expressed proteins between various conditions. Furthermore, protein–protein interaction network analysis was conducted using Cytoscape 3.9.0 software (21). Centrality analysis was conducted to identify the most important node with a high degree of interaction in the network. The functional enrichment analysis was conducted using the g:Profiler tool.

## Results

**Figure 1:**
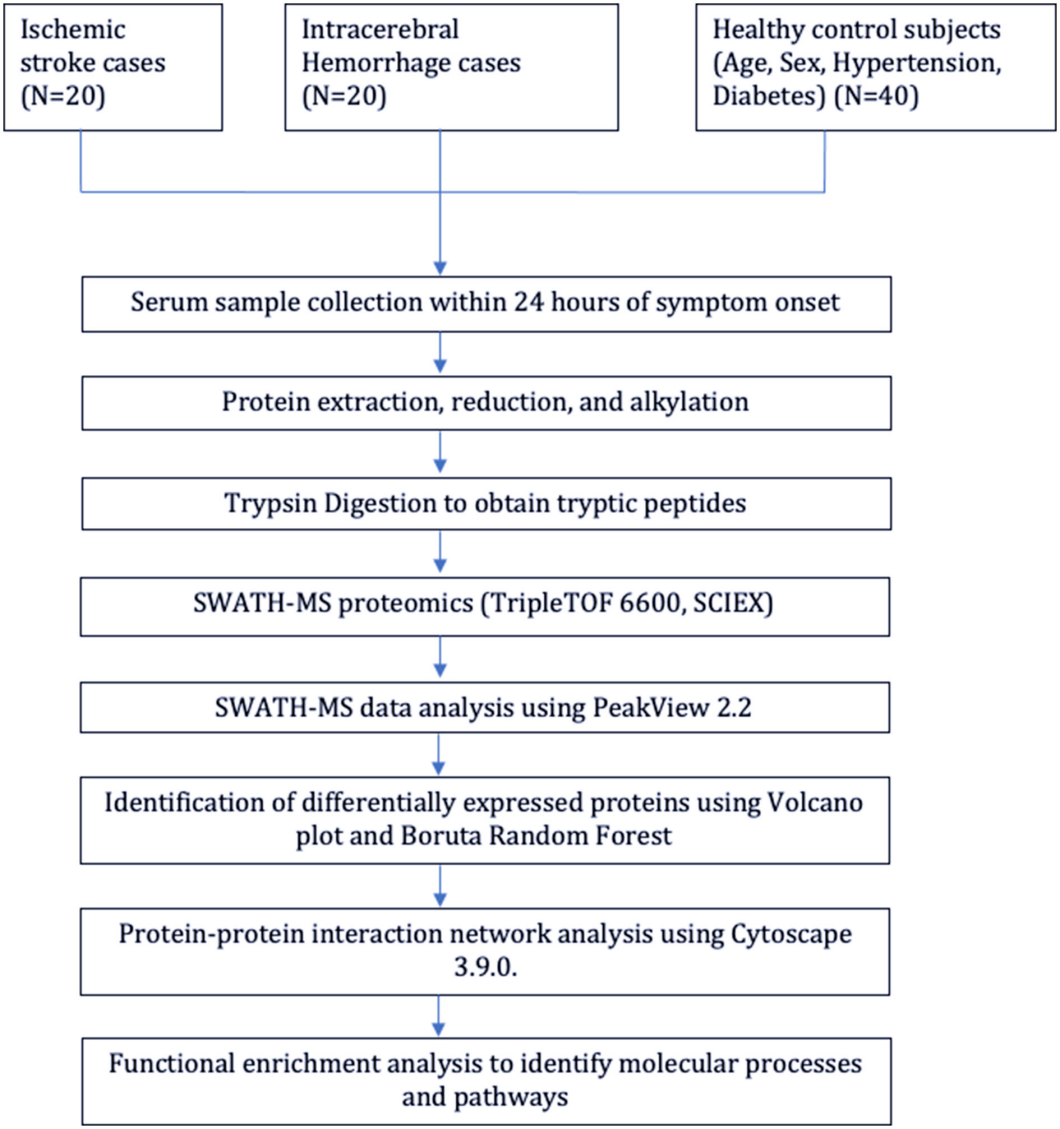
Study flow diagram.

Our study included 80 subjects; 40 stroke cases (20 IS and 20 ICH) were recruited within 24 h of symptom onset and age- (±2), sex-, hypertension-, and diabetes-matched 40 healthy control subjects. The mean age of IS, ICH, and control subjects was 52.85 ± 10.86, 47.60 ± 9.76, and 50.20 ± 10.64 years, respectively. Both stroke cases and healthy controls consisted of 25 (62.5%) males and 15 (37.5%) females, respectively. The mean blood sampling time (in h) from the symptom onset was 12.11 ± 6.23 in IS cases and 12.46 ± 6.68 in ICH cases (p=0.86). The study flow diagram is given in Figure 1. The baseline characteristics of the subjects included in our study are given in Table 1, and blood investigations are given in Supplementary Table 1.

**Table 1:** Baseline characteristics of acute stroke patients and healthy control subjects.

**S. No**	**Characteristics**	**IS patients (*N* = 20)**	**ICH patients (*N* = 20)**	***p-*value**	**Total stroke (*N* = 40)**	**No. of obs. (Controls)**	**Control subjects (*N* = 40)**	***P-*value**
1.	Age (years), Mean ± SD and Median (IQR)	52.85 ± 10.86, 53.5 (45.5–61.5)	47.60 ± 9.76, 48 (43–55.5)	0.12	50.22 ± 10.53, 49 (45–59.5)	40	50.20 ± 10.69, 48.5 (44.5–60)	Matched
2.	Male, *n* (%)	11 (55)	14 (70)	0.33	25 (62.5)	40	25 (62.5)	
3.	Female, *n* (%)	9 (45)	6 (30)		15 (37.5)	40	15 (37.5)	
4.	Blood sampling time from onset (in h.), Mean ± SD & Median (IQR)	12.11 ± 6.23, 11.5 (7.12–17)	12.46 ± 6.68, 12.58 (6.25–18.62)	0.86	12.28 ± 6.38, 12.58 (6.5–17.75)	-	-	-
5.	Time taken to reach hospital (in hrs.), Mean ± SD & Median (IQR)	4.21 ± 2.98, 3.87 (2–5)	6.41 ± 6.27, 3.75 (2.08–10.12)	0.16	5.31 ± 4.97, 3.87 (2–5.75)	-	-	-
6.	Ambulance as a mode of transport, *n* (%)	6 (30)	4 (20)	0.53	10 (25)	-	-	-
7.	Any surgical procedure, *n* (%)	2 (10)	5 (25)	0.21	7 (17.5)	-	-	-
**Risk factors for stroke**								
8.	Hypertension, *n* (%)	8 (40)	14 (70)	0.06	22 (55)	40	22 (55)	Matched
9.	Diabetes, *n* (%)	4 (20)	1 (5)	0.15	5 (12.5)	40	5 (12.5)	
10.	Dyslipidemia, n (%)	4 (20)	0 (0)	**0.03**	4 (10)	40	6 (15)	0.50
11.	Myocardial Infarction, *n* (%)	0	0	–	0	40	1 (2.5)	0.31
12.	Atrial Fibrillation, *n* (%)	0	0	–	0	40	1 (2.5)	0.31
13.	Angina Pectoris, *n* (%)	1 (5%)	0	0.31	1 (2.5)	30	1 (3.33)	0.83
14.	Migraine, *n* (%)	0	0	–	0	40	3 (7.50)	0.08
15.	Current Smoking, *n* (%)	9 (45)	10 (50)	0.75	19 (47.5)	40	7 (17.50)	**0.004**
17.	Alcohol Intake, *n* (%)	2 (10)	6 (30)	0.11	8 (20)	40	11 (27.5)	0.43
17.	No exercise, *n* (%)	18 (90)	17 (85)	0.63	35 (87.5)	39	11 (28.21)	**< 0.001**
18.	Sedentary lifestyle, *n* (%)	7 (35)	7 (35)	1.00	14 (35)	37	6 (16.22)	0.06
19.	Low Education, *n* (%)	13 (65)	15 (75)	0.49	28 (70)	39	12 (30.77)	**0.0005**
20.	Low socio-economic status, *n* (%)	9 (45)	11 (55)	0.53	20 (50)	40	0	**< 0.001**
21.	Obesity, *n* (%)	7 (35)	10 (50)	0.34	17 (42.5)	38	24 (63.16)	0.07
22.	Family history of stroke, *n* (%)	3 (15)	1 (5)	0.29	4 (10)	40	2 (5)	0.39
23.	Family history of hypertension, *n* (%)	10 (50)	6 (30)	0.20	16 (40)	40	12 (30)	0.44
24.	Family history of diabetes, *n* (%)	7 (35)	3 (15)	0.14	10 (25)	40	16 (40)	0.15
25.	Family history of heart attack, *n* (%)	4 (20)	3 (15)	0.68	7 (17.5)	40	12 (30)	0.19
**Vitals at admission**								
26.	SBP (mmHg), Mean ± SD & Median (IQR)	152.7 ± 35.71, 147 (127.5–175)	178 ± 35.29, 176 (150–214)	**0.03**	165.35 ± 37.31, 163 (137–190)	39	141.46 ± 24.55, 134 (121–155)	**0.001**
27.	DBP (mmHg), Mean ± SD & Median (IQR)	87.5 ± 17.27, 87 (80–95)	100.10 ± 17.00, 100 (90–110)	**0.02**	93.8 ± 18.08, 90 (82–104)	39	89.95 ± 15.90, 90 (78–98)	0.32

obs, observations; IS, ischemic stroke; ICH, intracerebral hemorrhage; SD, standard deviation; IQR, interquartile range; SBP, systolic blood pressure; DBP, diastolic blood pressure.

**Bold values**, p < 0.05.

### SWATH-MS to identify differential proteome in stroke cases and controls

Serum proteomic profiles were compared between 40 stroke (20 IS and 20 ICH) and 40 healthy controls using the SWATH-MS approach. From the high-pH fractionated peptide library for human serum proteins (obtained from SCIEX) comprising of 465 proteins, we could quantify 375 proteins at 1% peptide FDR between the stroke cases and control groups through SWATH-MS analysis. The total ion chromatogram (TIC) of all the 80 serum samples analyzed using the discovery-based SWATH-MS proteomics is given in Supplementary Figure 1. The batch variation observed in our samples due to the different run times was removed as depicted in the PCA plots in Supplementary Figure 2.

### Differentially expressed proteins between total stroke and healthy controls

**Figure 2:**
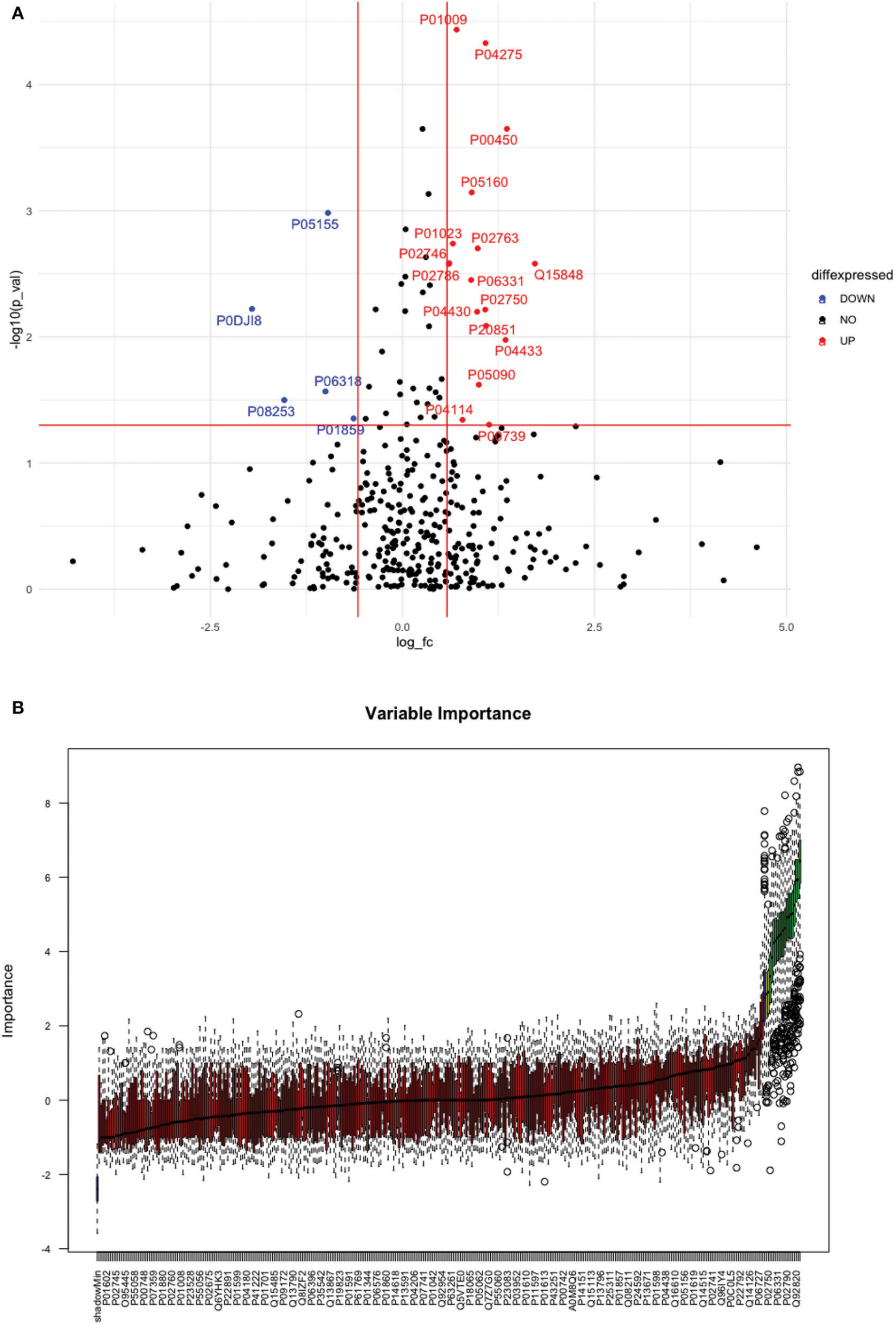
**(A)** Volcano plot depicting the log_2_ fold change on the x-axis and -log10 *p-*value on the y-axis for the upregulated and downregulated proteins in 40 total stroke cases compared to 40 healthy controls. The two vertical lines represent the threshold for log2 fold change (>0.58 and < −0.58), and the horizontal line represents the threshold for log10 *p-*value (>1.3). The red dots on the graph indicate the proteins which are significantly upregulated (log2 fold change>0.58; log10 *p-*value>1.3), and blue dots indicate the proteins which are significantly downregulated (log2 fold change < −0.58; log10 *p-*value>1.3). **(B)** Feature selection using the Boruta random forest depicting important features/proteins to differentiate 40 total stroke and 40 healthy controls. The blue bars on the graph indicate shadow features for minimum, average, and maximum shadow feature values. The red bars on the graph indicate the proteins which were rejected as irrelevant features, yellow bars indicate the proteins which were marked tentative as uncertain features, and green bars indicate the proteins which were marked confirmed as valid features and identified as important proteins after the Boruta random forest feature selection analysis.

**Figure 3:**
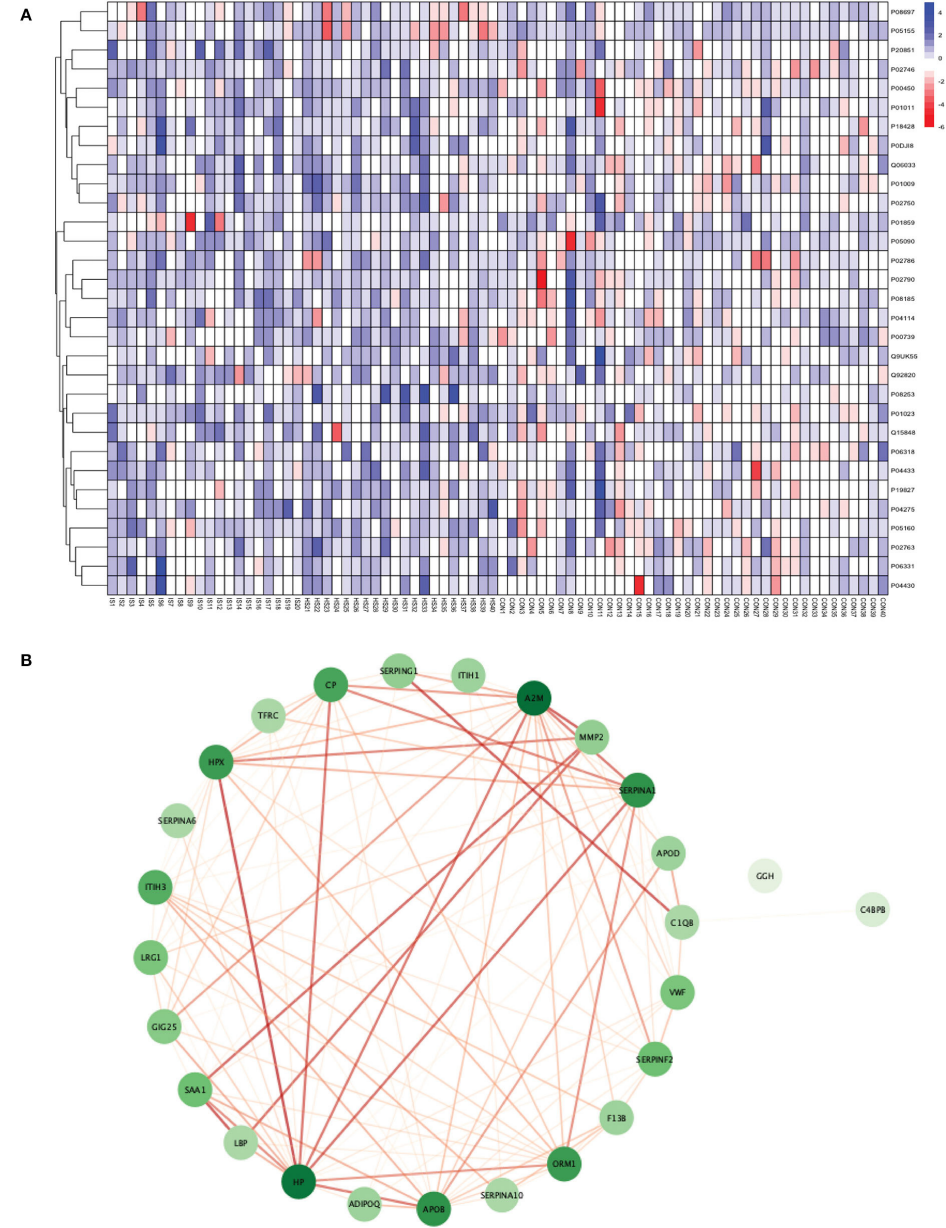
**(A)** Heatmap showing the distribution and expression pattern of 31 significantly differentially expressed proteins between total stroke and healthy controls. Each column represents a different subject, while each row represents the UniProt ID of different proteins. The blue squares represent upregulated, and the red squares represent downregulated proteins in total stroke compared to healthy controls. White squares represent no change in protein expression. **(B)** Protein–protein interaction network analysis of differentially expressed proteins between total stroke and healthy controls. The color of the nodes represents the level of degree of interaction between the proteins ranging from 0 to 19, with dark green representing a high degree of interaction (toward 19) and light green representing a low degree of interaction (toward zero). The color of edges represents the interaction score ranging from zero to one, with dark red edges representing an interaction score with high confidence (toward one) and light red edges representing an interaction score with low confidence (toward zero).

Between 40 stroke and 40 control subjects, 119 proteins were upregulated with a fold change of >1.5, and 72 were downregulated with a fold change of < 0.67 in total stroke cases compared to healthy controls. Using the fold change and *p-*value cutoffs, 22 proteins were significantly differentially expressed between total stroke and healthy controls. Seventeen proteins were significantly upregulated, while five were significantly downregulated in total stroke compared to healthy controls (Figure 2A). Using the Boruta random forest method, 19 proteins were identified as confirmed/tentative features (Figure 2B; Supplementary Table 2). Ten proteins (UniProt IDs: P00450, P01009, P04275, P05160, P05155, P02750, P02786, Q15848, P06318, and P06331) were common in both the fold change with *p-*value and the Boruta random forest criteria. Thus, after combining the distinctly expressed proteins using both approaches, 31 significantly differentially expressed proteins were identified between total stroke and control subjects within 24 h (Table 2). A heatmap of 31 significantly differentially expressed proteins showing the log_2_ fold change expression pattern between total stroke and controls is given in Figure 3A.

**Table 2:** List of significantly differentially expressed proteins between total stroke and healthy controls within 24 h of symptom onset using fold change with *p-*value and Boruta random forest feature selection criteria.

**S. No**	**UniProt ID**	**Protein name (Gene annotation)**	Fold change*	***P-*value**	**Boruta decision**
1	Q15848	Adiponectin (GN *=* ADIPOQ)	**3.30**	**0.003**	**Confirmed**
2	P00450	Ceruloplasmin (GN *=* CP)	**2.57**	**0.0002**	**Confirmed**
3	P04433	Ig kappa chain V-III region VG (Fragment)	**2.54**	**0.01**	Rejected
4	P00739	Haptoglobin-related protein (GN *=* HPR)	**2.18**	**0.04**	Rejected
5	P20851	C4b-binding protein beta chain (GN *=* C4BPB)	**2.13**	**0.008**	Rejected
6	P04275	von Willebrand factor (GN *=* VWF)	**2.12**	**< 0.001**	**Confirmed**
7	P02750	Leucine-rich alpha-2-glycoprotein (GN *=* LRG1)	**2.11**	**0.006**	**Tentative**
8	P05090	Apolipoprotein D (GN *=* APOD)	**1.99**	**0.02**	Rejected
9	P02763	Alpha-1-acid glycoprotein 1 (GN *=* ORM1)	**1.97**	**0.002**	Rejected
10	P04430	Ig kappa chain V-I region BAN	**1.96**	**0.006**	Rejected
11	P05160	Coagulation factor XIII B chain (GN *=* F13B)	**1.87**	**0.0007**	**Confirmed**
12	P06331	Ig heavy chain V-II region ARH-77	**1.86**	**0.0035**	**Confirmed**
13	P04114	Apolipoprotein B-100 (GN *=* APOB)	**1.72**	**0.045**	Rejected
14	P01009	Alpha-1-antitrypsin (GN *=* SERPINA1)	**1.63**	**< 0.001**	**Confirmed**
15	P01023	Alpha-2-macroglobulin (GN *=* A2M)	**1.58**	**0.002**	Rejected
16	P02746	Complement C1q subcomponent subunit B (GN *=* C1QB)	**1.53**	**0.003**	Rejected
17	P02786	Transferrin receptor protein 1 (GN *=* TFRC)	**1.52**	**0.003**	**Confirmed**
18	Q92820	Gamma-glutamyl hydrolase (GN *=* GGH)	1.28	**0.03**	**Confirmed**
19	Q06033	Inter-alpha-trypsin inhibitor heavy chain H3 (GN *=* ITIH3)	1.26	**0.0007**	**Confirmed**
20	P02790	Hemopexin (GN *=* HPX)	1.20	**0.004**	**Confirmed**
21	P18428	Lipopolysaccharide-binding protein (GN *=* LBP)	1.20	**0.0002**	**Confirmed**
22	P01011	Alpha-1-antichymotrypsin (GN *=* SERPINA3)	1.03	**0.001**	**Confirmed**
23	P08185	Corticosteroid-binding globulin (GN *=* SERPINA6)	0.99	**0.004**	**Confirmed**
24	P19827	Inter-alpha-trypsin inhibitor heavy chain H1 (GN *=* ITIH1)	0.98	0.06	**Confirmed**
25	Q9UK55	Protein Z-dependent protease inhibitor (GN *=* SERPINA10)	0.85	0.073	**Confirmed**
26	P08697	Alpha-2-antiplasmin (GN *=* SERPINF2)	0.78	**0.006**	**Confirmed**
27	P01859	Ig gamma-2 chain C region (GN *=* IGHG2)	**0.64**	**0.04**	Rejected
28	P05155	Plasma protease C1 inhibitor (GN *=* SERPING1)	**0.51**	**0.001**	**Confirmed**
29	P06318	Ig lambda chain V-VI region WLT	**0.50**	**0.03**	**Tentative**
30	P08253	72 kDa type IV collagenase (GN *=* MMP2)	**0.34**	**0.03**	Rejected
31	P0DJI8	Serum amyloid A-1 protein (GN *=* SAA1)	**0.26**	**0.006**	Rejected

^*^Fold change is a ratio representing the change of protein concentration between total stroke cases and healthy control subjects.

**Bold values**, Fold change >1.5 or < 0.67, p-value < 0.05 and confirmed/tentative in Boruta random forest.

Out of 31 proteins, 26 were successfully matched to proteins within the STRING database. The interaction network consisted of 26 nodes and 115 edges. Twenty-five proteins formed a highly connected network except for the GGH protein. Centrality analysis identified that APOB had the highest degree of interaction (DoI)= 19 with other proteins followed by haptoglobin (HP) (DoI = 18), APOB (DoI = 15), and SERPINA1 (DoI= 15). Eight protein–protein interactions in our network had an interaction score of more than 0.90, with the highest interaction score of 0.97 for HPX-HP followed by 0.944 for LBP-SAA1, 0.940 for SERPING1-C1QB, 0.937 for MMP2-A2M, and 0.92 for MMP2-SAA1 (Figure 3B).

**Figure 4:**
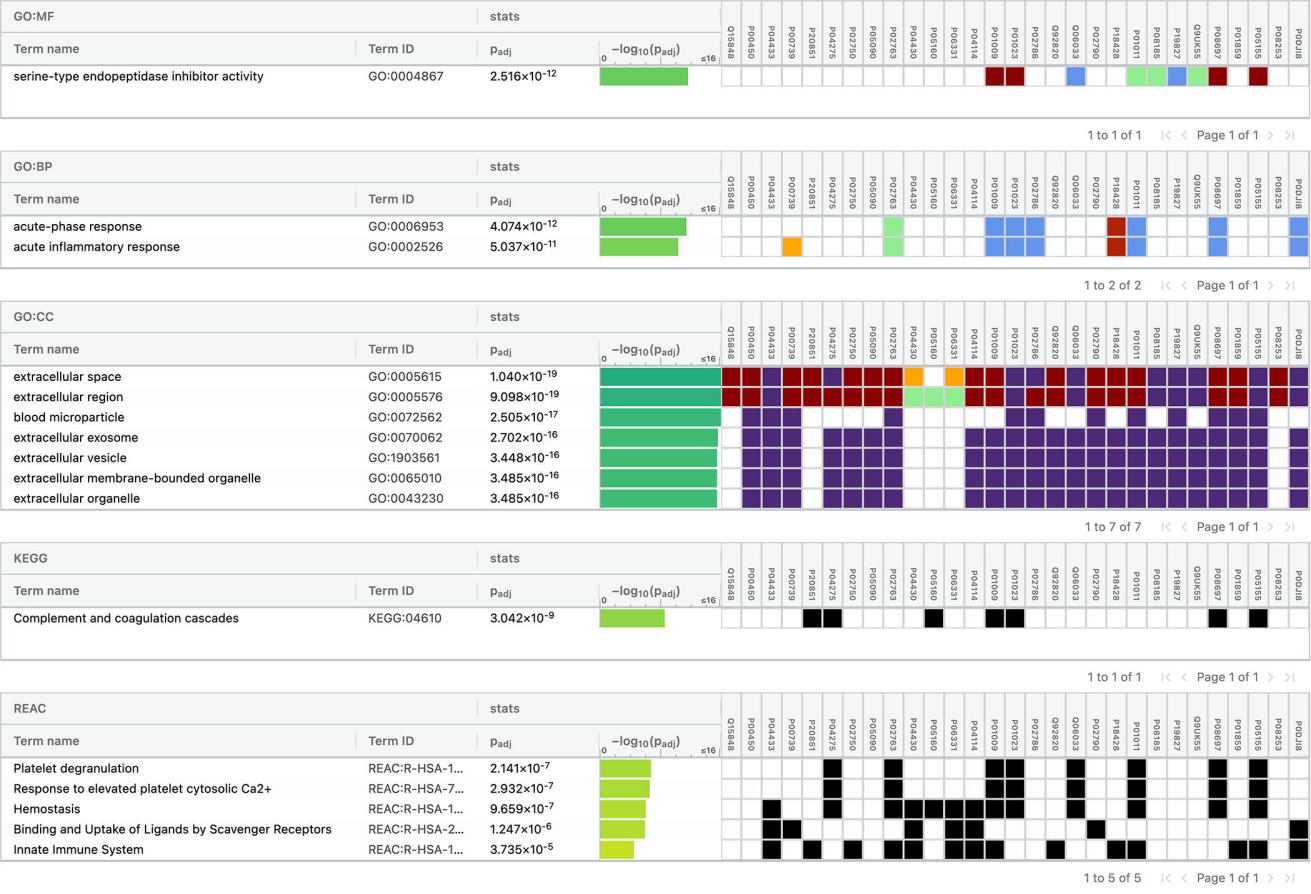
Functional enrichment analysis of differentially expressed proteins between total stroke and healthy controls.

Using Gene Ontology (GO) database, the top 10 cellular components, molecular interactions, or biological processes involved are as follows: serine-type endopeptidase inhibitor activity, acute phase response, acute inflammatory response, extracellular space, extracellular region, blood microparticle, extracellular exosome, extracellular vesicle, extracellular membrane-bounded organelle, and extracellular organelle. One Kyoto Encyclopedia of Genes and Genomes (KEGG) pathway, namely complement and coagulation cascades, was identified to be significantly involved. Using the Reactome database, the top five pathways involved are as follows: platelet degranulation, response to elevated platelet cytosolic Ca2+, hemostasis, binding and uptake of ligands by Scavenger Receptors, and innate immune system (Figure 4).

### Differentially expressed proteins between ischemic stroke and healthy controls

**Figure 5:**
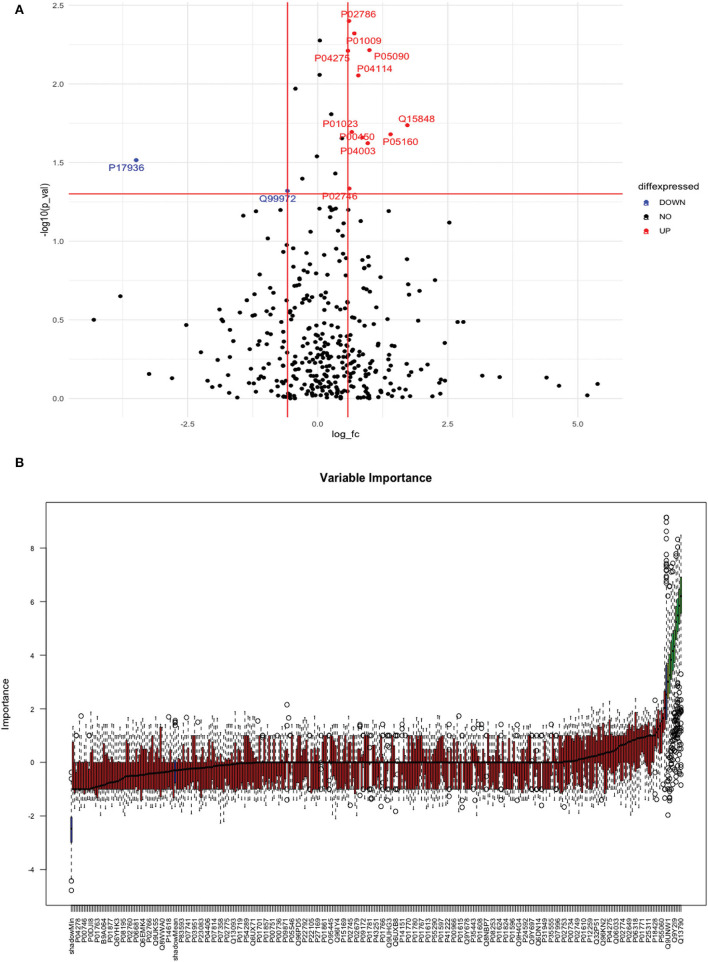
**(A)** Volcano plot depicting the log2 fold change on the x-axis and –log10 *p-*value on the y-axis for the upregulated and downregulated proteins in 20 IS cases compared to 20 healthy controls. **(B)** Feature selection using the Boruta random forest depicting important features/proteins to differentiate 20 IS and 20 healthy controls.

**Figure 6:**
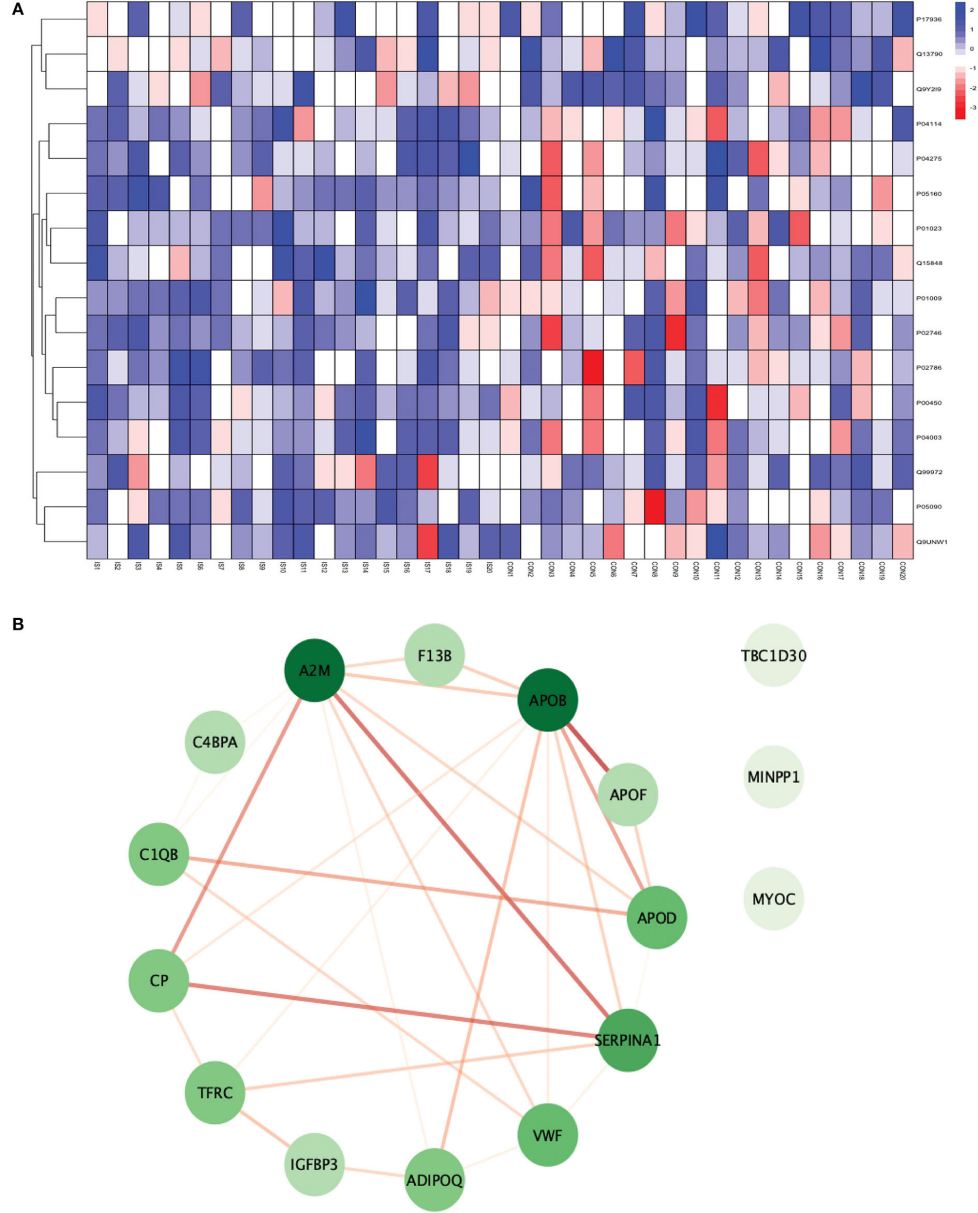
**(A)** Heatmap showing the distribution and expression pattern of 16 significantly differentially expressed proteins between IS and healthy controls. **(B)** Protein–protein interaction network analysis of differentially expressed proteins between IS and healthy controls.

Between 20 IS and 20 controls, 118 proteins were upregulated, and 72 were downregulated in IS cases compared to control subjects. Using the fold change and *p-*value criteria, 13 proteins were significantly differentially expressed, wherein 11 were significantly upregulated while two were significantly downregulated in IS compared to controls (Figure 5A). Using the Boruta random forest method, nine more proteins were identified as confirmed/tentative features (Figure 5B; Supplementary Table 3). Six proteins (UniProt IDs: P04114, P01023, P01009, P02786, P05090, and Q99972) were common using both approaches. Finally, 16 distinct proteins were identified using the above two criteria which were significantly differentially expressed between IS and healthy controls within 24 h (Table 3). A heatmap of 16 significantly differentially expressed proteins showing the log_2_ fold change expression pattern between IS and healthy controls is given in Figure 6A.

**Table 3:** List of significantly differentially expressed proteins between ischemic stroke and healthy controls using fold change with *p-*value and Boruta random forest feature selection criteria.

**S. No**	**UniProt ID**	**Protein name (Gene annotation)**	Fold change*	***P-*value**	**Boruta selection**
1	Q15848	Adiponectin (GN *=* ADIPOQ)	**3.30**	**0.02**	Rejected
2	P05160	Coagulation factor XIII B chain (GN *=* F13B)	**2.64**	**0.02**	Rejected
3	P05090	Apolipoprotein D (GN *=* APOD)	**1.99**	**0.01**	**Confirmed**
4	P04003	C4b-binding protein alpha chain (GN *=* C4BPA)	**1.95**	**0.02**	Rejected
5	P00450	Ceruloplasmin (GN *=* CP)	**1.81**	**0.02**	Rejected
6	P04114	Apolipoprotein B-100 (GN *=* APOB)	**1.72**	**0.01**	**Confirmed**
7	P01009	Alpha-1-antitrypsin (GN *=* SERPINA1)	**1.63**	**0.005**	**Confirmed**
8	P01023	Alpha-2-macroglobulin (GN *=* A2M)	**1.58**	**0.02**	**Confirmed**
9	P02746	Complement C1q subcomponent subunit B (GN *=* C1QB)	**1.53**	**0.04**	Rejected
10	P02786	Transferrin receptor protein 1 (GN *=* TFRC)	**1.52**	**0.04**	**Confirmed**
11	P04275	von Willebrand factor (GN *=* VWF)	**1.50**	**0.01**	Rejected
12	Q13790	Apolipoprotein F (GN *=* APOF)	1.38	**0.02**	**Confirmed**
13	Q9UNW1	Multiple inositol polyphosphate phosphatase 1 (GN *=* MINPP1)	1.18	0.07	**Confirmed**
14	Q9Y2I9	TBC1 domain family member 30 (GN *=* TBC1D30)	0.74	**0.01**	**Confirmed**
15	Q99972	Myocilin (GN *=* MYOC)	**0.66**	**0.04**	**Confirmed**
16	P17936	Insulin-like growth factor-binding protein 3 (GN *=* IGFBP3)	**0.09**	**0.03**	Rejected

^*^Fold change is a ratio representing the change of protein concentration between IS cases and healthy control subjects.

**Bold values:** Fold change >1.5 or < 0.67, p-value < 0.05 and confirmed/tentative in Boruta random forest.

All the 16 proteins were successfully matched within the STRING database. The interaction network analysis identified 16 nodes and 29 edges. Thirteen out of 16 proteins formed a highly connected network with other proteins, whereas three proteins (TBC1D30, MINPP1, and MYOC) remained disconnected from the network. Centrality analysis identified that APOB and SERPINA1 had the highest DoI of 9 with other proteins, followed by vWF (DoI = 6), APOD (DoI = 5), and TFRC (DoI = 5). Seven protein–protein interactions in our network had an interaction score of more than 0.70 with the highest protein–protein interaction score of 0.950 for APOB-APOF followed by 0.888 for A2M-SERPINA1, 0.857 for CP-SERPINA1, and 0.806 for CP-A2M (Figure 6B).

**Figure 7:**
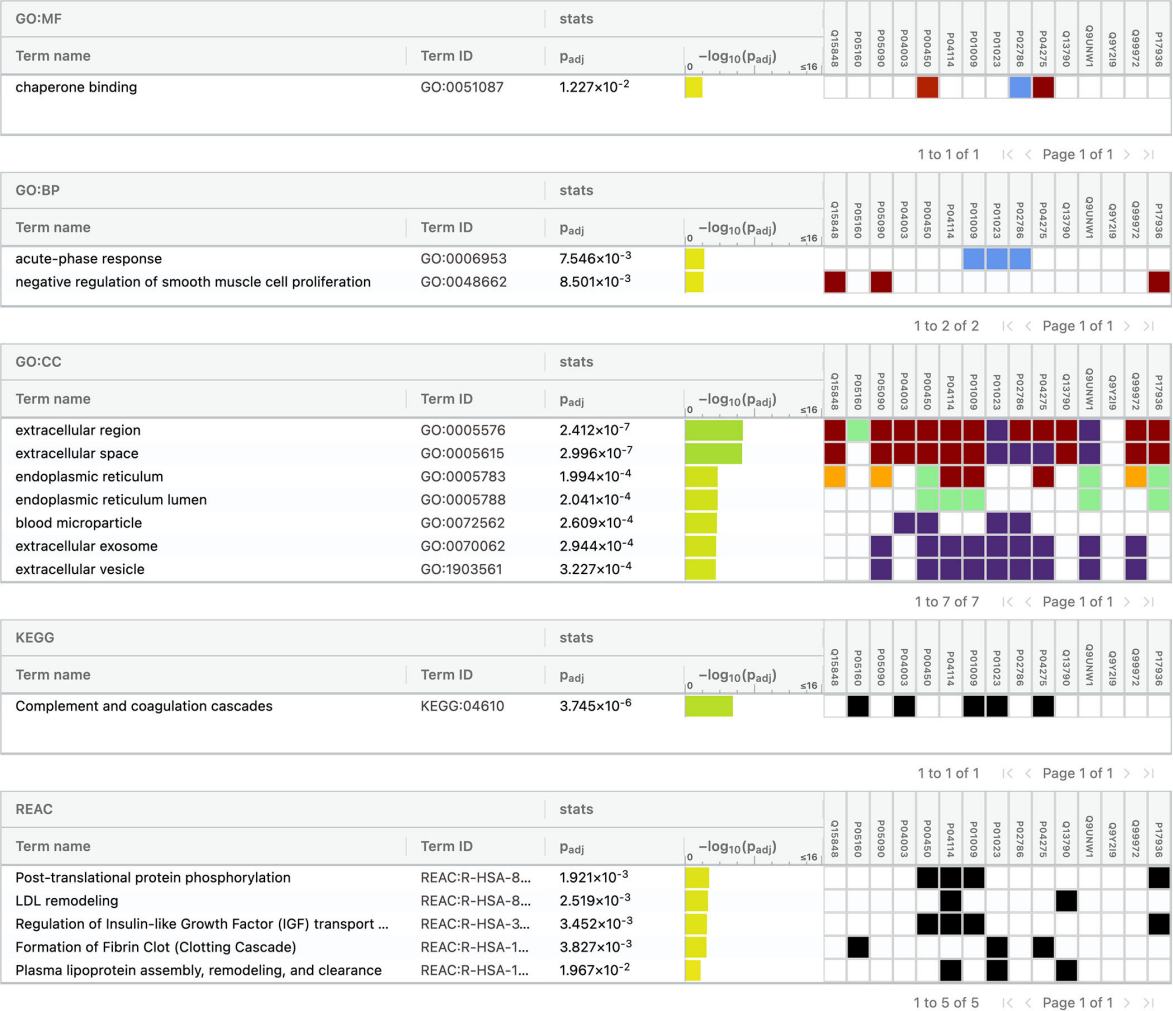
Functional enrichment analysis of differentially expressed proteins between ischemic stroke and healthy controls.

For conducting the functional enrichment analysis of significantly differentially expressed proteins identified between IS and controls, the top 10 cellular components were selected in the GO database, including chaperone binding, acute phase response, negative regulation of smooth muscle cell proliferation, extracellular region, extracellular space, endoplasmic reticulum, endoplasmic reticulum lumen, blood microparticle, extracellular exosome, and extracellular vesicle. The only KEGG pathway that was found to be significantly associated was complement and coagulation cascades. When the enrichment analysis was done using the Reactome database, the top five pathways observed were formation of fibrin clot (clotting cascade), regulation of IGF transport and uptake by IGFBPs, post-translational protein phosphorylation, LDL remodeling, and plasma lipoprotein assembly, remodeling, and clearance (Figure 7).

### Differentially expressed proteins between intracerebral hemorrhage and healthy controls

**Figure 8:**
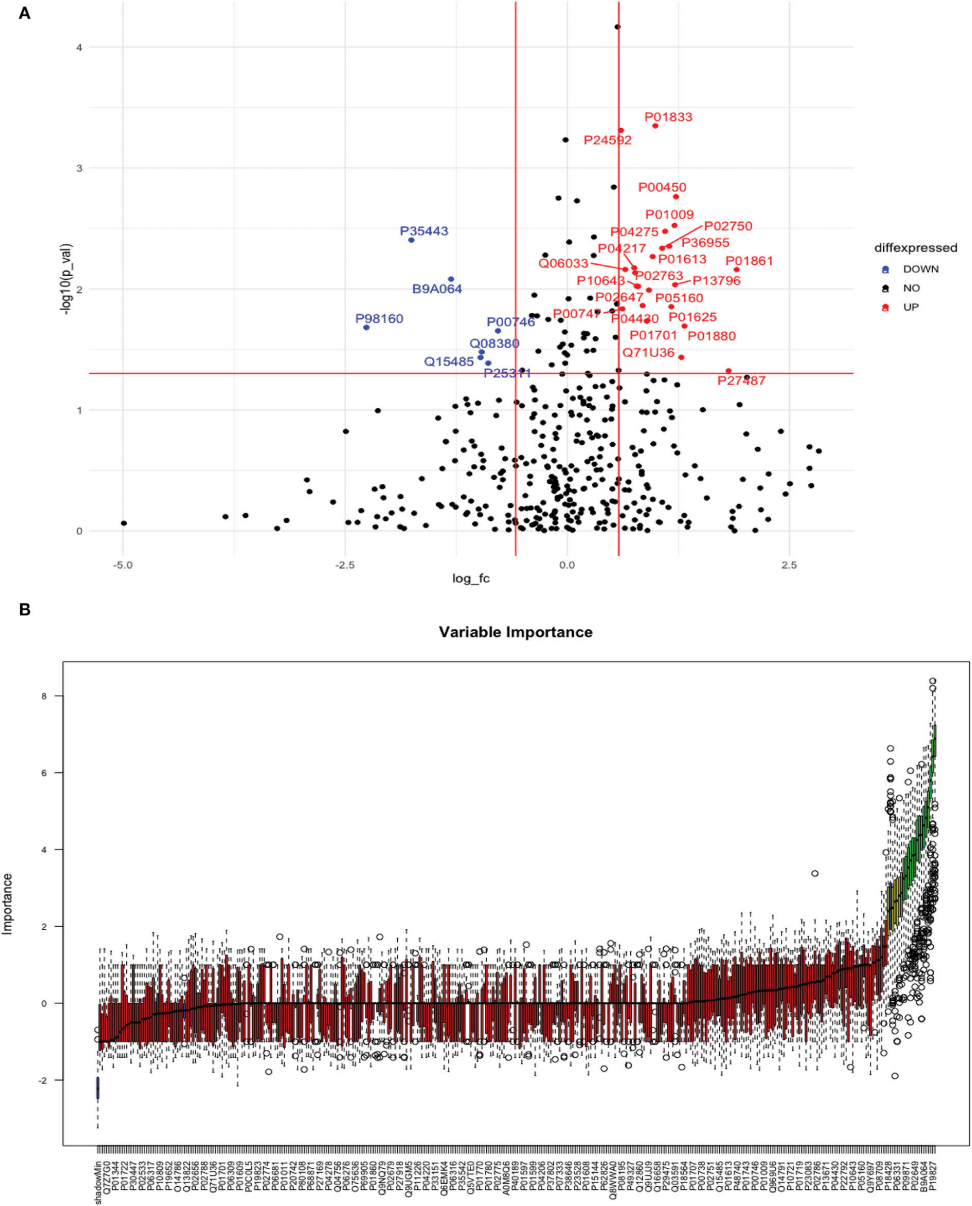
**(A)** Volcano plot depicting the log2 fold change on the x-axis and –log10 *p-*value on the y-axis for the upregulated and downregulated proteins in 20 ICH cases compared to 20 healthy controls. **(B)** Feature selection using the Boruta random forest depicting important features/proteins to differentiate 20 ICH and 20 healthy controls.

**Figure 9:**
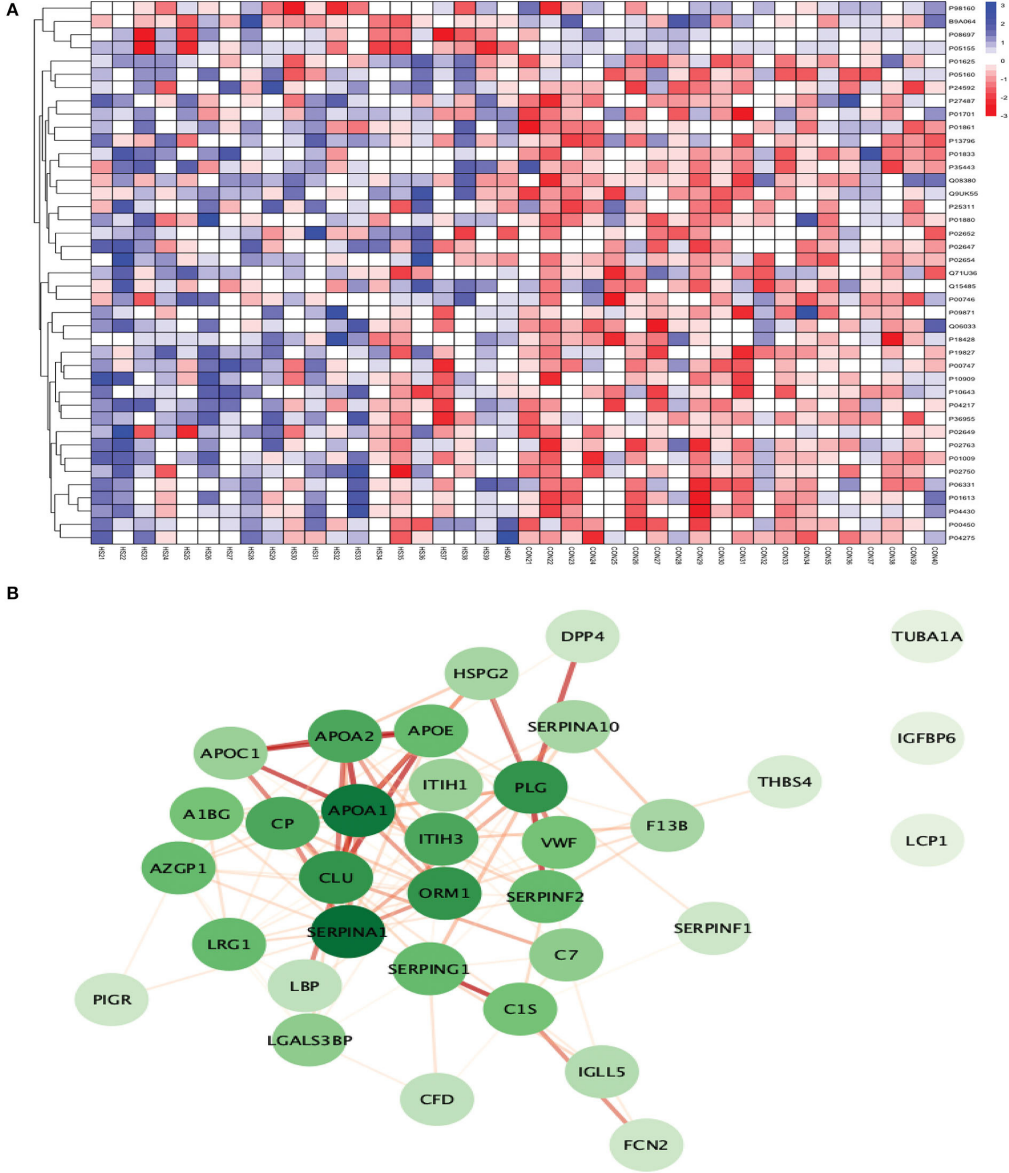
**(A)** Heatmap showing the distribution and expression pattern of 41 significantly differentially expressed proteins between ICH and healthy controls. **(B)** Protein–protein interaction network analysis of differentially expressed proteins between ICH and healthy controls.

Between 20 ICH and 20 controls, 102 proteins were upregulated while 83 were downregulated in ICH cases compared to control subjects. Using the fold change and *p-*value criteria, 30 proteins were significantly differentially expressed; 23 significantly upregulated and seven significantly downregulated in ICH cases compared to healthy controls (Figure 8A). Using the Boruta random forest method, 21 more proteins were further identified as confirmed/tentative features (Figure 8B; Supplementary Table 4). Ten proteins (UniProt IDs: P00450, P04275, Q06033, P04217, P36955, B9A064, P02750, P01833, P35443, and P24592) were common using both the above-mentioned criteria for protein selection. Thus, after combining the distinct proteins using both the criteria, we identified 41 proteins that significantly differentially expressed ICH from healthy controls within 24 h (Table 4). A heatmap of 41 significantly differentially expressed proteins showing the log_2_ fold change expression pattern between ICH and healthy controls is given in Figure 9A.

**Table 4:** List of significantly differentially expressed proteins between intracerebral hemorrhage and healthy controls using fold change with *p-*value and Boruta random forest feature selection criteria.

**S. No**	**UniProt ID**	**Protein name (Gene annotation)**	Fold change*	***P-*value**	**Boruta decision**
1	P01861	Ig gamma-4 chain C region (GN *=* IGHG4)	**3.75**	**0.01**	Rejected
2	P27487	Dipeptidyl peptidase 4 (GN *=* DPP4)	**3.52**	**0.05**	Rejected
3	P01880	Ig delta chain C region (GN *=* IGHD)	**2.49**	**0.02**	Rejected
4	Q71U36	Tubulin alpha-1A chain (GN *=* TUBA1A)	**2.43**	**0.04**	Rejected
5	P00450	Ceruloplasmin (GN *=* CP)	**2.33**	**0.00**	**Confirmed**
6	P13796	Plastin-2 (GN *=* LCP1)	**2.32**	**0.01**	Rejected
7	P01009	Alpha-1-antitrypsin (GN *=* SERPINA1)	**2.31**	**0.00**	Rejected
8	P02649	Apolipoprotein E (GN *=* APOE)	**2.30**	**0.20**	**Confirmed**
9	P01625	Ig kappa chain V-IV region Len	**2.25**	**0.01**	Rejected
10	P36955	Pigment epithelium-derived factor (GN *=* SERPINF1)	**2.21**	**0.004**	**Confirmed**
11	P04275	von Willebrand factor (GN *=* VWF)	**2.15**	**0.003**	**Tentative**
12	P02750	Leucine-rich alpha-2-glycoprotein (GN *=* LRG1)	**2.10**	**0.005**	**Confirmed**
13	P01833	Polymeric immunoglobulin receptor (GN *=* PIGR)	**1.99**	**0.0004**	**Confirmed**
14	P09871	Complement C1s subcomponent (GN *=* C1S)	**1.96**	**0.06**	**Confirmed**
15	P01613	Ig kappa chain V-I region Ni	**1.95**	**0.01**	Rejected
16	P05160	Coagulation factor XIII B chain (GN *=* F13B)	**1.89**	**0.01**	Rejected
17	P01701	Ig lambda chain V-I region NEW	**1.86**	**0.02**	Rejected
18	P04430	Ig kappa chain V-I region BAN	**1.80**	**0.01**	Rejected
19	P10643	Complement component C7 (GN *=* C7)	**1.74**	**0.01**	Rejected
20	P02647	Apolipoprotein A-I (GN *=* APOA1)	**1.72**	**0.01**	Rejected
21	P02763	Alpha-1-acid glycoprotein 1 (GN *=* ORM1)	**1.70**	**0.01**	Rejected
22	P04217	Alpha-1B-glycoprotein (GN *=* A1BG)	**1.69**	**0.01**	**Tentative**
23	Q06033	Inter-alpha-trypsin inhibitor heavy chain H3 (GN *=* ITIH3)	**1.57**	**0.01**	**Tentative**
24	P00747	Plasminogen (GN *=* PLG)	**1.54**	**0.01**	Rejected
25	P24592	Insulin-like growth factor-binding protein 6 (GN *=* IGFBP6)	**1.52**	**0.0005**	**Confirmed**
26	P19827	Inter-alpha-trypsin inhibitor heavy chain H1 (GN *=* ITIH1)	1.48	**< 0.001**	**Confirmed**
27	Q9UK55	Protein Z-dependent protease inhibitor (GN *=* SERPINA10)	1.44	**0.001**	**Confirmed**
28	P08697	Alpha-2-antiplasmin (GN *=* SERPINF2)	1.42	**0.02**	**Confirmed**
29	P10909	Clusterin (GN *=* CLU)	1.27	**0.02**	**Confirmed**
30	P06331	Ig heavy chain V-II region ARH-77	1.23	**0.004**	**Tentative**
31	P18428	Lipopolysaccharide-binding protein (GN *=* LBP)	1.23	**0.01**	**Tentative**
32	P05155	Plasma protease C1 inhibitor (GN *=* SERPING1)	1.01	**0.004**	**Confirmed**
33	P02654	Apolipoprotein C-I (GN *=* APOC1)	0.99	**0.001**	**Confirmed**
34	P02652	Apolipoprotein A-II (GN *=* APOA2)	0.94	**0.03**	**Confirmed**
35	P00746	Complement factor D (GN *=* CFD)	**0.58**	**0.02**	Rejected
36	P25311	Zinc-alpha-2-glycoprotein (GN *=* AZGP1)	**0.54**	**0.04**	Rejected
37	Q08380	Galectin-3-binding protein (GN *=* LGALS3BP)	**0.51**	**0.03**	Rejected
38	Q15485	Ficolin-2 (GN *=* FCN2)	**0.51**	**0.04**	Rejected
39	B9A064	Immunoglobulin lambda-like polypeptide 5 (GN *=* IGLL5)	**0.40**	**0.01**	**Confirmed**
40	P35443	Thrombospondin-4 (GN *=* THBS4)	**0.30**	**0.004**	**Confirmed**
41	P98160	Basement membrane-specific heparan sulfate proteoglycan core protein (GN *=* HSPG2)	**0.21**	**0.02**	Rejected

^*^Fold change is a ratio representing the change of protein concentration between ICH cases and healthy control subjects.

**Bold values:** Fold change >1.5 or < 0.67, p-value < 0.05 and confirmed/tentative in Boruta random forest.

Out of 41 proteins, 34 successfully matched within the STRING database. The interaction network analysis identified 34 nodes and 125 edges. Except for three proteins (TUBA1A, IGFBP6, and LCP1), the interaction network of the remaining 31 proteins was highly connected with each other. SERPINA1 had the highest DoI of 18 with other proteins followed by APOA1 (DoI = 17), CLU (DoI =14), PLG (DoI = 14), and ORM1 (DoI = 14) after conducting the centrality analysis. Twelve protein–protein interactions in our network had an interaction score of more than 0.90 with the highest protein–protein interaction score of 0.999 for APOA1-APOA2 and SERPING1-C1S followed by 0.998 for PLG-SERPINF2, 0.997 for APOA1-APOE, and 0.995 for APOA1-CLU and APOE-APOC1 (Figure 9B).

**Figure 10:**
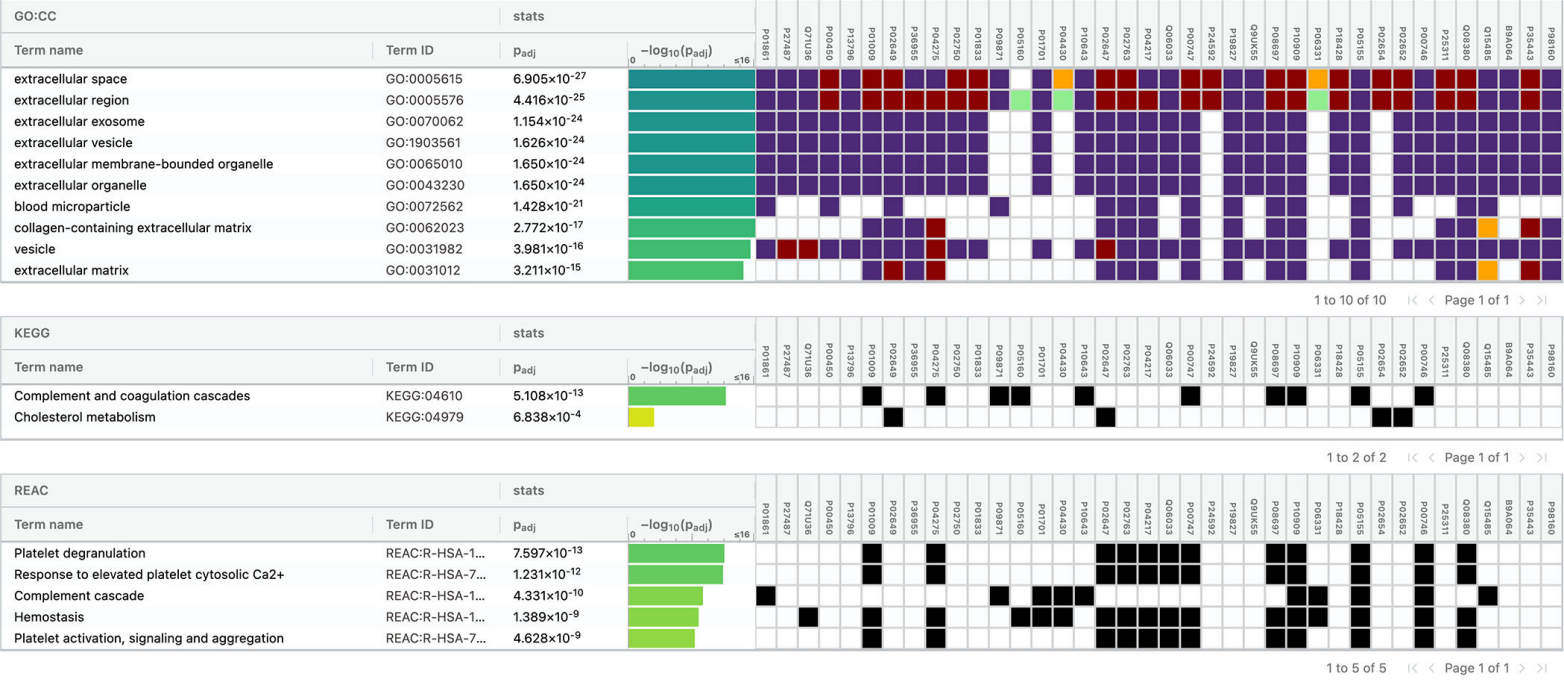
Functional enrichment analysis of differentially expressed proteins between intracerebral hemorrhage and healthy controls.

The top 10 cellular components and biological processes when analyzed using the GO database involved are as follows: extracellular space, extracellular exosome, extracellular region, extracellular vesicle, extracellular membrane-bounded organelle, extracellular organelle, blood microparticle, collagen-containing extracellular matrix, vesicle, and extracellular matrix. Using the KEGG database, we observed that two pathways were significantly involved: complement and coagulation cascades and cholesterol metabolism. The top five pathways identified using the Reactome database were platelet degranulation, response to elevated platelet cytosolic Ca2+, complement cascade, hemostasis, platelet activation, signaling, and aggregation. Complement cascade was a common pathway identified in KEGG and Reactome databases (Figure 10).

## Discussion

A stroke, if left untreated, results in the loss of 1.9 million neurons per min after its onset (22). Therefore, rapid diagnosis of stroke is critical to initiate stroke type-specific treatment and prevent large-scale brain damage. In this discovery phase study, we identified several differentially expressed proteins in stroke and its subtypes that elucidated key pathological processes involved in the acute phase of IS and ICH. Our study identified four proteins (ceruloplasmin, SERPINA1, vWF, and F13B) that commonly differentiated total stroke, IS, and ICH from healthy control subjects. To the best of our knowledge, this is the first label-free proteomic study that identified blood biomarkers for the diagnosis of stroke within 24 h of symptom onset. A list of proteomic studies conducted till now for the identification of diagnostic biomarkers in stroke is given in the Supplementary Table 5.

### Protein biomarkers identified between total stroke and healthy controls

We identified 31 significantly differentially expressed proteins between total stroke (IS + ICH) and healthy controls within 24 h. Twenty-five proteins formed a highly connected network, with APOB having the highest DoI. The most common significant pathways involved complement and coagulation cascades, immune-related processes, acute phase response, acute inflammatory response, hemostasis, and pathways related to extracellular space and matrix. In the Malicek et al. (9) study, they identified 12 significantly differentially expressed proteins between seven stroke and two control subjects in plasma. The stroke subjects were recruited within an average of 7 days (1–15 days) of symptom onset. Only two proteins (ITIH3 and LBP) were commonly differentially expressed in our study when compared to Malicek et al. (9). Another proteomic study conducted by Allard et al. (23) in plasma samples utilized the SELDI approach and identified four differentially expressed proteins (Apo C-1, Apo C-III, serum amyloid A, and antithrombin-III fragment) between 21 total stroke (IS = 11, ICH = 10) and 21 healthy controls recruited within 72 h. Of these four proteins, serum amyloid A was also differentially expressed in our study and was significantly downregulated in total stroke (fold change= 0.26) compared to the control group.

### Protein biomarkers identified between ischemic stroke and healthy controls

Between IS and healthy controls, our study identified 16 proteins within 24 h. The interaction network for 13 out of 16 proteins was highly connected. APOB and SERPINA1 had the highest DoI within the network. The most common significant pathways/processes associated with these proteins included complement and coagulation cascade, acute phase response, blood microparticle, clot formation, and pathways including extracellular region. A few studies in the past have used the proteomic approach to identify diagnostic biomarkers in IS compared to healthy controls, but most of these studies recruited IS patients beyond the 24-h time window. In a study published last year by Malicek et al. (9) on plasma samples, four proteins were significantly differentially expressed between three IS and two controls using a label-free proteomic approach. The IS subjects in this exploratory study were recruited within an average duration of 7 days (1–15 days) from symptom onset. No protein was commonly expressed upon comparing their results with our study. The difference in the protein expression profile between our studies could be attributed to the small sample size and longer blood sample collection time in the Malicek et al. study (9). Another recent study by Lee et al. (7) on serum samples used a similar approach of discovery-based SWATH-MS proteomics and identified 163 differential proteins with more than 2-fold change in 20 IS patients recruited within 10 days of symptom onset compared to 20 healthy controls. After applying the FDR-corrected *p-*values, they identified 13 significant biomarker candidates. C4BPA was the only common protein that was differentially expressed (upregulated in IS in both studies) in our study and in Lee et al. (7). The same authors conducted another SWATH-MS proteomic study to identify serum biomarkers related to coagulation cascade between 18 IS cases recruited within 7 days and 16 healthy controls. (8). They identified 60 upregulated (fold change >1.5) and 50 downregulated (fold change < 1/1.5) proteins in IS compared to controls out of which four proteins (prothrombin, plasminogen, fibrinogen alpha chain, and histidine-rich glycoprotein) related to coagulation cascade were finally selected, none of which were identified in our study. Another study by Qin et al. (10) on plasma samples recruited 40 IS patients with large vessel occlusion (LVO) within 7 days of symptom onset and 20 healthy controls. They identified seven differentially expressed proteins with a fold change of >1.2 or < 0.83 between the two groups using the iTRAQ labeling-based proteomic approach. No protein was commonly expressed between our study and Qin et al. (10). Therefore, the differential proteins identified in our study within 24 h were vastly different from the ones identified in Lee et al. (7) within 10 days, Lee et al. (8) and Qin et al. (10) within 7 days of symptom onset. When comparing our results with the other three studies, these contrasting findings provide crucial insights into the differences in the expression level of protein markers in the acute phase of stroke (within 24 h) compared to 7–10 days after the stroke onset.

The only proteomic study conducted on stroke patients in the Indian population by Sharma et al. (11) quantified 389 proteins using the iTRAQ labeling approach between pooled serum samples of 20 IS and 20 healthy controls in their discovery phase and identified 60 proteins with a difference of 1.5-fold or greater between the two groups. They observed that 25 proteins were more abundant, while 35 were less abundant in IS cases compared to controls. Using the *p-*value cutoff, they observed 23 significantly differentially expressed proteins. Compared to their study, we obtained three times more (180 proteins) differential proteins in our study after applying the 1.5-fold cutoff criteria. Adiponectin and vWF were two proteins that were significantly differentially expressed in both the studies, and both were upregulated in IS patients compared to controls. However, the study by Sharma et al. (11) did not mention the time duration for blood sample collection from IS subjects.

Besides blood biomarkers, proteomic studies between IS and healthy controls have also been conducted on other biofluids. The platelet activation response was assessed in a study by Cevik et al. (24) in nine IS cases recruited within 24 h and equal number of control subjects. Using the UPLC-ESI-q-TOF-MS proteomic approach, they identified 83 statistically significant (*p* < 0.05) proteins in the platelets between the two groups. Two proteins (ceruloplasmin and SERPINA1) were commonly differentially expressed between our study and Cevik et al. (24); however, both were not statistically significant in the Cevik et al. study (24). Both proteins were upregulated in our study, while both were downregulated in Cevik et al. (24) in IS cases compared to control subjects. This difference between the expression pattern of the two proteins might be due to the different biofluids used to assess the biomarker levels in both studies. Future comparative studies between serum and platelet proteomic markers are required to validate these findings. Wang et al. (25) recently conducted a urinary proteomic study using the DIA approach between 35 carotid artery stenosis (CAS) patients and 18 healthy controls. They did not mention the timing of sample collection in CAS patients. They identified 194 significantly differentially expressed proteins in urine samples between the two groups (fold change >1.5 and < 0.67 with *p* < 0.05), of which only myocilin was commonly expressed in our study. However, myocilin was downregulated in our study in contrast to Wang et al. (25), where it was upregulated. Since, Wang et al. recruited only IS patients with CAS, the difference in the expression pattern might be due to the different subtype of patient populations recruited in both the studies. Another urinary proteomic analysis was conducted by Dawson et al. (26) in a sample of 65 IS/TIA cases and 41 control subjects with urine samples collected within 24 h. Using the capillary electrophoresis-MS approach, they identified 35 statistically significant biomarkers between the two groups. Only ceruloplasmin was the statistically significant protein which was common between Dawson et al. and our study. A study conducted by Brea et al. (27) recruited 11 IS patients and an equal number of control subjects and isolated endothelial progenitor cell colonies within 7 days of symptom onset. They identified four differentially expressed proteins (endoplasmic reticulum protein-29, CdC-42, elongation factor-2, and peroxiredoxin-1) using the 2DE proteomic approach.

### Protein biomarkers identified between intracerebral hemorrhage and healthy controls

We identified 41 significantly differentially expressed proteins between ICH and controls within 24 h. Thirty-four proteins formed a highly connected network, and the DoI was strongest for SERPINA1. The most common significant pathways underlying proteins that differentiated ICH from controls included pathways related to the extracellular region, platelet degranulation, complement and coagulation cascade, cholesterol metabolism, and hemostasis. The literature on proteomic studies for the identification of diagnostic biomarkers for ICH is scarce. In the recent study by Malicek et al. (9) on plasma samples, 14 proteins were significantly differentially expressed between four ICH cases recruited within an average of 7 days (1–15 days) and two control subjects using a label-free proteomic approach. Plasminogen, inter-alpha-trypsin inhibitor heavy chain H3 (ITIH3), and lipopolysaccharide-binding protein (LBP) were three proteins that were commonly expressed in Malicek et al. (9) and our study. Lopez et al. (28) used the multiple reaction monitoring-based targeted proteomic approach on plasma samples and identified that Apo C-I individually and in combination with Apo A-II differentiated 26 ICH from 31 control subjects recruited within 7 days of symptom onset. Both the proteins were also confirmed in our study using the Boruta random forest method as important features for differentiating ICH from controls. Using the targeted metabolomic approach, Zhang et al. (29) used metabolites and recently identified two metabolic markers, i.e., 20-OH-LTB4 and arachidonic acid which differentiated 42 ICH cases from 65 control subjects recruited within 5 days. Our study identified novel protein biomarkers not discovered previously using a proteomic approach (apart from Apo C-I and Apo A-II) and provided crucial insights into the pathophysiology of ICH in acute stages.

## Future directions

This discovery phase study provides crucial insights into the pathophysiology of stroke and its subtypes. It provides a potential list of candidate protein markers to explore and new methodological strategies, including the use of label-free high-throughput proteomics for conducting biomarker research in stroke. The label-free SWATH-MS proteomic approach used in this study provides relative protein expression with high sensitivity and selectivity. It also has the capacity to maintain a high throughput, allowing it to evaluate many samples in a short duration of time with minimal operator intervention. However, extensive work still needs to be done before these biomarkers can be implemented in the clinical settings. A point-of-care test needs to be developed for rapid assessment of these biomarkers in hospital settings. Future studies must validate our findings in a large cohort of stroke patients using either standard immunoassays or targeted proteomic approaches. They must identify the sensitivity, specificity, and positive and negative predictive values of these biomarkers for diagnosing stroke. Studies should further aim at collecting blood samples in the hyperacute phase of stroke within 3–4.5 h, which is the clinically acceptable time window for administering thrombolytic therapy. A temporal profile depicting the expression pattern of these biomarkers over the 24-h period is also urgently warranted in stroke patients.

## Limitations

We conducted a pilot/discovery phase study; thus, the findings were only exploratory. We obtained relative quantification values for each protein. Therefore, our findings warrant validation in a large cohort using absolute quantification approaches. Since we collected serum samples in our study, the proteins highlighting the significant role of platelet granulation in stroke might account for some false positives due to the activation of platelets in the serum samples.

## Conclusion

Our discovery phase exploratory study identified a list of potential protein biomarker candidates for the diagnosis of acute stroke and highlighted significant molecular pathways associated with different stroke subtypes. The results of our study could serve as a platform for conducting future validation studies. These potential biomarker candidates need to be validated in studies using either standard immunoassays or targeted proteomic approach in a large cohort of stroke patients to investigate their diagnostic performance.

## Data availability statement

The datasets presented in this study can be found in online repositories. The names of the repository/repositories and accession number(s) can be found below: https://www.ebi.ac.uk/pride/archive/projects/PXD032917.

## Ethics statement

The studies involving human participants were reviewed and approved by the Local Institutional Ethics Committee of AIIMS, New Delhi (Ref. No. IECPG-395/28.09.2017). The patients/participants provided their written informed consent to participate in this study.

## Author contributions

DV conceptualized the idea of this research topic, helped design the clinical methodology, and supervised each step of execution of this study. SM primarily conducted each step of this study ranging from blood sample collection, processing, proteomic experimentation, statistical and proteomic data analysis, results interpretation, and manuscript writing. SSG supervised the proteomic experimentation and its data analysis. SM and PS conducted the proteomic experiments and data analysis. DB contributed in conducting the proteomic experimentations in the study. MN contributed in patient sample collection and processing. AK helped in statistical data analysis. DV, PA, AKS, AKP, DM, and KP aided in patient recruitment for this study. All authors contributed to the article and approved the submitted version.

## Funding

This study was supported in part by the AIIMS Intramural Research Grant (F. No. 8-762/A-762/2019/RS).

## Conflict of interest

The authors declare that the research was conducted in the absence of any commercial or financial relationships that could be construed as a potential conflict of interest.

## Publisher's note

All claims expressed in this article are solely those of the authors and do not necessarily represent those of their affiliated organizations, or those of the publisher, the editors and the reviewers. Any product that may be evaluated in this article, or claim that may be made by its manufacturer, is not guaranteed or endorsed by the publisher.
