# Supplementary material for: Blood-based protein biomarkers for the diagnosis of acute stroke: A discovery-based SWATH-MS proteomic approach
Source: Front Neurol. 2022 Sep 27;13:989856. doi: 10.3389/fneur.2022.989856 (PMC9552908; doi:10.3389/fneur.2022.989856)
Supplement: Supplementary file 1 [file Table_1.DOCX]

**
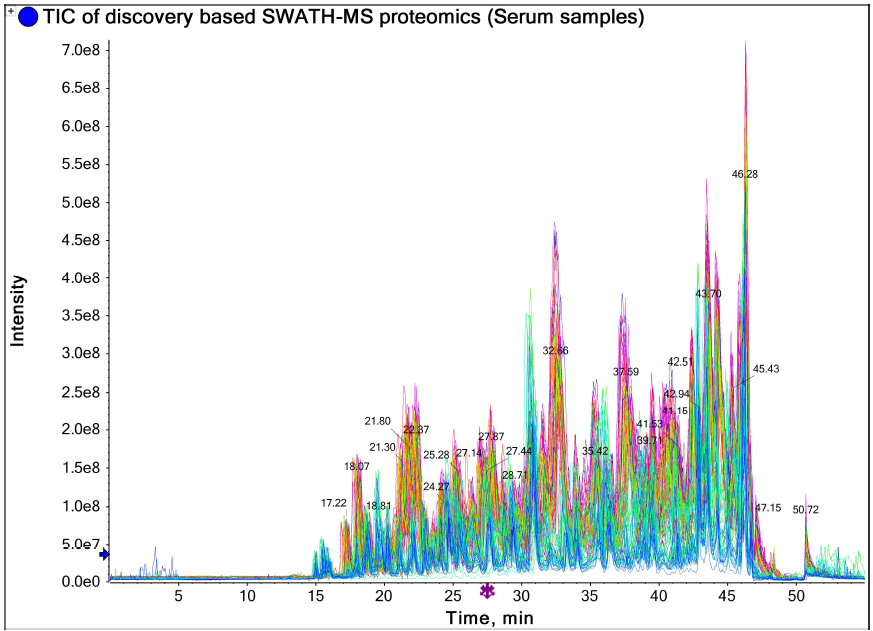
**

**Supplementary Figure 1:** Total Ion Chromatogram depicting the elution profile of 40 stroke cases and 40 control subjects for the discovery-based SWATH-MS proteomics.

**
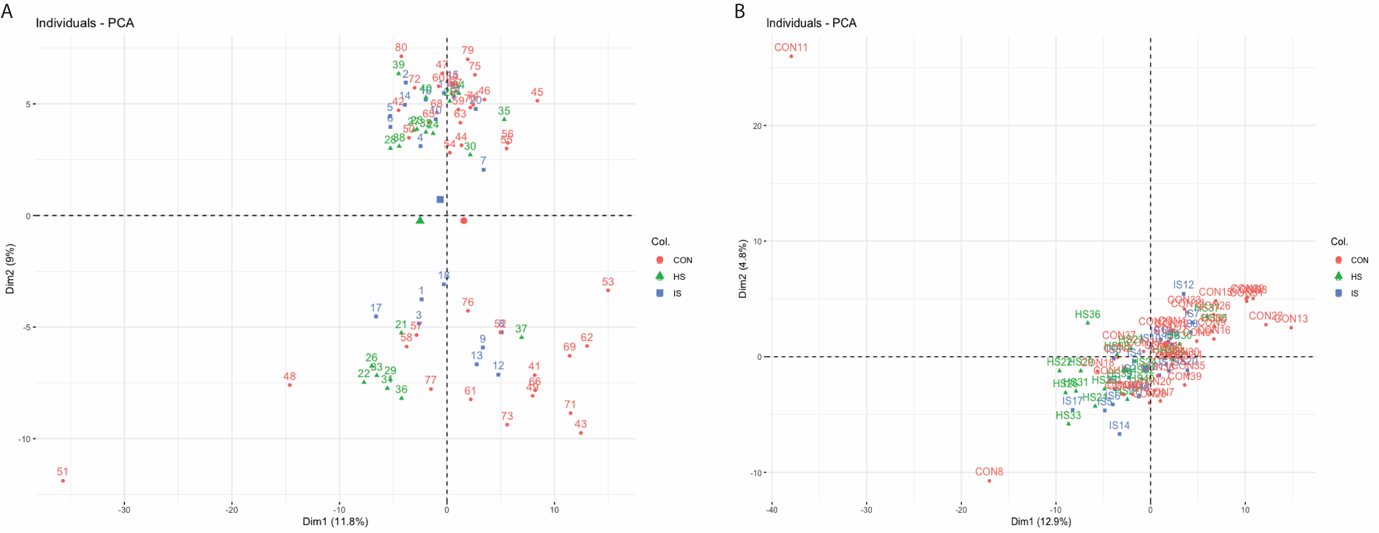
**

**Supplementary Figure 2: (a)** Principal Component Analysis plot of the 80 serum samples before batch correction. **(b)** Principal Component Analysis plot of the 80 serum samples after applying the batch correction. CON: Healthy Controls; HS: Hemorrhagic Stroke; IS: Ischemic Stroke.

**Supplementary Table 1:** Blood investigations of acute stroke patients recruited in the discovery phase study

| **S. No** | **Characteristics** | **No. of obs. (IS)** | **IS patients (N=20)** | **No. of obs. (ICH)** | **ICH patients (N=20)** | **p-value** | **No. of obs. (Total Stroke)** | **Total stroke (N=40)** |
| --- | --- | --- | --- | --- | --- | --- | --- | --- |
| 1. | RBS (mg/dl), Mean ± SD & Median (IQR) | 19 | 150.26 ± 74.49,  120 (112-152) | 16 | 136.62 ± 86.13,  120 (93.5-142) | 0.62 | 35 | 144.03 ± 79.11,  120 (100-145) |
| 2. | HbA1C (%), Mean ± SD & Median (IQR) | 16 | 6.73 ± 1.95,  5.9 (5.6-7.5) | 10 | 5.91 ± 0.60,  5.9 (5.4-6.5) | 0.21 | 26 | 6.41 ± 1.61,  5.9 (5.5-6.7) |
| 3. | Homocysteine (µM/l), Mean ± SD & Median (IQR) | 16 | 24.42 ± 17.12,  18.59 (12.88-32.47) | 3 | 63.59 ± 2.44,  65 (60.77-65) | **0.001** | 19 | 30.60 ± 21.45,  20.47 (13.11-54.58) |
| 4. | Total Cholesterol (mg/dl), Mean ± SD & Median (IQR) | 19 | 189.79 ± 68.52,  171 (151-211) | 11 | 173.36 ± 38.18,  179 (151-199) | 0.47 | 30 | 183.77 ± 59.01,  179 (151-202) |
| 5. | LDL (mg/dl), Mean ± SD & Median (IQR) | 19 | 128.49 ± 51.37,  122 (94-140) | 11 | 116.44 ± 33.05,  116 (94-150) | 0.49 | 30 | 124.07 ± 45.27,  119 (94-140) |
| 6. | HDL (mg/dl), Mean ± SD & Median (IQR) | 19 | 40.33 ± 11.30,  40 (33-46) | 11 | 44.01 ± 10.25,  44.1 (35-54) | 0.38 | 30 | 41.68 ± 10.90,  41 (35-46) |
| 7. | VLDL (mg/dl), Mean ± SD & Median (IQR) | 19 | 22.30 ± 10.60,  19 (15-26) | 11 | 16.21 ± 4.17,  15 (14-19) | 0.08 | 30 | 20.07 ± 9.20,  18.08 (14.16-23) |
| 8. | TG (mg/dl), Mean ± SD & Median (IQR) | 19 | 135.51 ± 64.32,  137 (84-170) | 11 | 126.78 ± 44.31,  118 (90.8-158) | 0.69 | 30 | 132.31 ± 57.12,  128.5 (87-169) |
| 9. | Hemoglobin (g/dl), Mean ± SD & Median (IQR) | 19 | 13.57 ± 1.87,  13.7 (11.7-14.8) | 17 | 14.07 ± 2.36,  14.2 (12.3-15.2) | 0.49 | 36 | 13.81 ± 2.10,  14.05 (12.1-15.04) |
| 10. | TLC (10^3^/µL), Mean ± SD & Median (IQR) | 19 | 9123.68 ± 2955.27,  9050 (7500-11690) | 17 | 8584.99 ± 3490.83,  8400 (6600-9800) | 0.62 | 36 | 8869.30 ± 3183.81,  8800 (7300-9945) |
| 11. | Platelets (10^3^/µL), Mean ± SD & Median (IQR) | 19 | 167.84 ± 71.33,  162 (106-191) | 17 | 158.07 ± 78.89,  130 (110-191) | 0.70 | 36 | 163.23 ± 74.07,  143 (108-191) |
| 12. | Neutrophils (%), Mean ± SD & Median (IQR) | 19 | 69.00 ± 13.10,  68.7 (61.2-79.8) | 17 | 74.80 ± 13.02,  78.3 (68.2-84.7) | 0.19 | 36 | 71.74 ± 13.20,  72.7 (63.85-82) |
| 13. | Eosinophils (%), Mean ± SD & Median (IQR) | 19 | 2.10 ± 1.49,  1.5 (1.2-3.1) | 17 | 2.40 ± 2.37,  1.3 (0.7-3.9) | 0.65 | 36 | 2.24 ± 1.93,  1.5 (0.85-3.5) |
| 14. | Basophils (%), Mean ± SD & Median (IQR) | 19 | 1.86 ± 1.89,  1 (0.5-2.6) | 17 | 2.70 ± 3.94,  1.4 (0.4-2.7) | 0.41 | 36 | 2.26 ± 3.02,  1.05 (0.45-2.65) |
| 15. | Lymphocytes (%), Mean ± SD & Median (IQR) | 19 | 21.42 ± 10.07,  19.8 (12.4-28.9) | 17 | 16.40 ± 8.33,  14.1 (9.2-21.9) | 0.11 | 36 | 19.05 ± 9.51,  19.4 (10.85-26.9) |
| 16. | Monocytes (%), Mean ± SD & Median (IQR) | 19 | 5.60 ± 3.70,  4.8 (3.9-6.5) | 17 | 3.69 ± 2.52,  2.7 (2.1-4.9) | 0.08 | 36 | 4.70 ± 3.30,  4.55 (2.5-5.75) |
| 17. | T3 (ng/dl), Mean ± SD & Median (IQR) | 18 | 79.85 ± 41.53,  87.33 (69.79-112.53) | 11 | 72.14 ± 43.54,  76.79 (55.46-95.15) | 0.64 | 29 | 76.93 ± 41.70,  78.97 (67.23-102.9) |
| 18. | T4 (μg /dl), Mean ± SD & Median (IQR) | 18 | 6.98 ± 2.31,  7.25 (5.4-8.6) | 11 | 7.40 ± 1.49,  7.52 (6.4-8.1) | 0.60 | 29 | 7.14 ± 2.02,  7.4 (5.5-8.4) |
| 19. | TSH (µIU/ml), Mean ± SD & Median (IQR) | 17 | 12.15 ± 35.67,  1.93 (1.31-6.04) | 11 | 1.84 ± 1.70,  0.94 (0.84-3.11) | 0.35 | 28 | 8.10 ± 27.95,  1.66 (0.93-3.11) |
| 20. | PT (seconds), Mean ± SD & Median (IQR) | 18 | 12.37 ± 1.39,  (12.15-11.1-13.2) | 19 | 12.24 ± 1.27,  11.8 (10.9-13) | 0.77 | 37 | 12.30 ± 1.32,  11.9 (11.1-13) |
| 21. | INR, Mean ± SD & Median (IQR) | 18 | 1.06 ± 0.11,  1.06 (0.97-1.11) | 19 | 1.06 ± 0.11,  1.04 (0.97-1.11) | 0.89 | 37 | 1.06 ± 0.10,  1.06 (0.97-1.11) |
| 22. | Vitamin B12 (pg/ml), Mean ± SD & Median (IQR) | 17 | 358.84 ± 205.07,  322 (262-397) | 8 | 289.37 ± 239.07,  278 (140.5-328.5) | 0.46 | 25 | 336.68 ± 214.02,  311 (222-357) |
| 23. | Vitamin D (ng/ml), Mean ± SD & Median (IQR) | 7 | 8.64 ± 4.56,  8.78 (4-12.8) | 5 | 5.71 ± 2.49,  4.61 (4.56-5.29) | 0.22 | 12 | 7.42 ± 3.98,  6.59 (4.28-10.4) |
| 24. | ESR (mm/hr), Mean ± SD & Median (IQR) | 9 | 23.67 ± 22.10,  15 (8-26) | 7 | 19.43 ± 9.83,  22 (10-25) | 0.64 | 16 | 21.81 ± 17.43,  22 (9-26) |
| 25. | Urea (mg%), Mean ± SD & Median (IQR) | 20 | 24.66 ± 8.35,  25 (18.5-32) | 18 | 26.96 ± 10.08,  25.99 (18-37) | 0.45 | 38 | 25.75 ± 9.16,  25.99 (18-34) |
| 26. | Creatinine (mg%), Mean ± SD & Median (IQR) | 20 | 0.84 ± 0.29,  0.85 (0.6-0.96) | 19 | 0.91 ± 0.42,  0.8 (0.7-1.3) | 0.53 | 39 | 0.87 ± 0.36,  0.8 (0.6-1) |
| 27. | Potassium (mM/L), Mean ± SD & Median (IQR) | 20 | 4.47 ± 0.65,  4.38 (3.96-4.99) | 19 | 4.12 ± 0.50,  4.06 (3.96-4.38) | 0.07 | 39 | 4.30 ± 0.60,  4.2 (3.96-4.59) |
| 28. | Sodium (mM/L), Mean ± SD & Median (IQR) | 20 | 138.18 ± 4.00,  137.5 (135-141.4) | 19 | 140.97 ± 4.66,  141.5 (137.6-144) | **0.05** | 39 | 139.54 ± 4.50,  139.2 (136-142.1) |
| 29. | ALP (I.U.), Mean ± SD & Median (IQR) | 19 | 104.79 ± 44.79,  93 (71-131) | 13 | 109.92 ± 66.70,  91 (74-115) | 0.79 | 32 | 106.87 ± 53.79,  92 (73-123) |
| 30. | Calcium (mg%), Mean ± SD & Median (IQR) | 19 | 8.92 ± 0.50,  9 (8.6-9.07) | 13 | 8.76 ± 0.83,  9 (8.32-9.37) | 0.48 | 32 | 8.86 ± 0.65,  9 (8.54-9.31) |
| 31. | SGOT (I.U.), Mean ± SD & Median (IQR) | 19 | 31.49 ± 18.31,  24 (21-33) | 13 | 35.88 ± 19.68,  29 (26-40) | 0.52 | 32 | 33.28 ± 18.70,  26.5 (23-40) |
| 32. | SGPT (I.U.), Mean ± SD & Median (IQR) | 19 | 22.18 ± 6.87,  23 (16-28) | 13 | 35.35 ± 25.52,  28 (18-47) | **0.04** | 32 | 27.53 ± 17.96,  23 (17.5-29.5) |
| 33. | Total Bilirubin (mg%), Mean ± SD & Median (IQR) | 20 | 0.74 ± 0.52,  0.62 (0.44-0.93) | 18 | 0.71 ± 0.23,  0.69 (0.6-0.9) | 0.86 | 38 | 0.73 ± 0.41,  0.67 (0.5-0.9) |
| 34. | Total Protein (gm%), Mean ± SD & Median (IQR) | 19 | 7.00 ± 0.55,  7.1 (6.7-7.3) | 12 | 6.77 ± 1.06,  7.15 (5.91-7.45) | 0.43 | 31 | 6.91 ± 0.78,  7.1 (6.6-7.4) |
| 35. | Albumin (gm%), Mean ± SD & Median (IQR) | 19 | 4.03 ± 0.37,  3.9 (3.8-4.3) | 12 | 3.94 ± 0.60,  4 (3.6-4.45) | 0.63 | 31 | 3.99 ± 0.46,  3.9 (3.8-4.3) |
| 36. | Globulin (gm%), Mean ± SD & Median (IQR) | 19 | 2.99 ± 0.56,  2.92 (2.7-3.2) | 12 | 2.82 ± 0.59,  2.95 (2.5-3.3) | 0.45 | 31 | 2.92 ± 0.57,  2.92 (2.59-3.2) |

**Abbreviations**: IS: Ischemic Stroke; ICH: Intracerebral Hemorrhage; RBS: Random Blood Sugar; LDL: Low-Density Lipoprotein; HDL: High-Density Lipoprotein; VLDL: Very Low-Density Lipoprotein; TG: Triglyceride; TLC: Total Leucocyte Count; PT: Prothrombin Time; INR: International Normalized Ratio; TSH: Thyroid Stimulating Hormone; ESR: Erythrocyte Sedimentation Rate; SGOT: Serum Glutamic Oxaloacetic Transaminase; SPGT: Serum Glutamic Pyruvic Transaminase; ALP: Alkaline Phosphatase; SD: Standard Deviation; IQR: Interquartile Range; Obs.: Observations.

**Bold values:** p<0.05.

**Supplementary Table 2**: Results of the confirmed/tentative attributes selected using the Boruta feature selection process between total stroke and healthy control subjects

| **Attributes (UniProt ID)** | **Mean Imp** | **Median Imp** | **Min Imp** | **Max Imp** | **Norm Hits** | **Decision** |
| --- | --- | --- | --- | --- | --- | --- |
| P00450 | 4.96 | 5.03 | 1.01 | 7.26 | 0.91 | Confirmed |
| P01009 | 3.81 | 3.84 | 0.87 | 6.72 | 0.75 | Confirmed |
| P02790 | 4.79 | 4.92 | -0.03 | 7.49 | 0.87 | Confirmed |
| P19827 | 3.25 | 3.33 | -1.89 | 5.59 | 0.63 | Confirmed |
| P04275 | 6.34 | 6.43 | 2.04 | 8.84 | 0.97 | Confirmed |
| P01011 | 4.23 | 4.29 | 0.46 | 6.70 | 0.83 | Confirmed |
| P05160 | 4.36 | 4.42 | -0.08 | 7.10 | 0.83 | Confirmed |
| Q06033 | 4.45 | 4.56 | -0.09 | 7.29 | 0.85 | Confirmed |
| P08697 | 4.89 | 5.00 | 0.24 | 7.12 | 0.89 | Confirmed |
| P05155 | 4.89 | 4.94 | 0.70 | 7.58 | 0.90 | Confirmed |
| P02750 | 2.89 | 2.91 | -0.06 | 5.27 | 0.53 | Tentative |
| P08185 | 4.39 | 4.47 | -1.11 | 7.11 | 0.86 | Confirmed |
| P02786 | 4.56 | 4.63 | -0.03 | 8.21 | 0.88 | Confirmed |
| Q9UK55 | 5.87 | 5.93 | 1.42 | 8.96 | 0.95 | Confirmed |
| P18428 | 5.18 | 5.25 | 0.88 | 8.59 | 0.92 | Confirmed |
| Q15848 | 4.17 | 4.24 | 0.14 | 6.65 | 0.79 | Confirmed |
| Q92820 | 5.86 | 5.93 | 1.57 | 8.18 | 0.95 | Confirmed |
| P06318 | 2.81 | 2.85 | -0.06 | 4.96 | 0.51 | Tentative |
| P06331 | 4.28 | 4.37 | 0.38 | 6.52 | 0.85 | Confirmed |

**Abbreviations**: Imp- Importance measure computed over multiple iterations; Mean Imp- the mean of Imp; Median Imp- the median of Imp; Min Imp- the minimum of Imp; Max Imp- the maximum of Imp; Norm Hits- the number of hits normalized to number of importance source runs.

**Supplementary Table 3**: Results of the confirmed/tentative attributes selected using the Boruta feature selection process between IS and healthy control subjects

| **Attributes (UniProt ID)** | **Mean Imp** | **Median Imp** | **Min Imp** | **Max Imp** | **Norm Hits** | **Decision** |
| --- | --- | --- | --- | --- | --- | --- |
| P04114 | 4.07 | 4.16 | -0.82 | 7.43 | 0.73 | Confirmed |
| P01023 | 5.71 | 5.85 | -0.42 | 8.33 | 0.90 | Confirmed |
| P01009 | 5.38 | 5.48 | -0.13 | 8.32 | 0.88 | Confirmed |
| P02786 | 3.76 | 3.83 | -1.00 | 7.30 | 0.72 | Confirmed |
| P05090 | 5.12 | 5.24 | -0.73 | 7.78 | 0.85 | Confirmed |
| Q13790 | 6.08 | 6.20 | -0.85 | 8.48 | 0.92 | Confirmed |
| Q9UNW1 | 2.96 | 3.01 | -1.97 | 6.21 | 0.58 | Tentative |
| Q9Y2I9 | 4.28 | 4.33 | -0.28 | 6.62 | 0.78 | Confirmed |
| Q99972 | 3.22 | 3.25 | -1.43 | 6.58 | 0.61 | Confirmed |

**Abbreviations**: Imp- Importance measure computed over multiple iterations; Mean Imp- the mean of Imp; Median Imp- the median of Imp; Min Imp- the minimum of Imp; Max Imp- the maximum of Imp; Norm Hits- the number of hits normalized to number of importance source runs.

**Supplementary Table 4**: Results of the confirmed/tentative attributes selected using the Boruta feature selection process between ICH and healthy control subjects within 24 hours

| **Attributes (UniProt ID)** | **Mean Imp** | **Median Imp** | **Min Imp** | **Max Imp** | **Norm Hits** | **Decision** |
| --- | --- | --- | --- | --- | --- | --- |
| P00450 | 5.00 | 5.10 | 1.57 | 6.71 | 0.94 | Confirmed |
| P19827 | 6.34 | 6.42 | 2.43 | 8.39 | 0.99 | Confirmed |
| P04275 | 2.75 | 2.80 | -0.37 | 5.34 | 0.59 | Tentative |
| P09871 | 3.25 | 3.32 | 0.71 | 5.44 | 0.73 | Confirmed |
| Q06033 | 2.60 | 2.64 | -1.89 | 4.90 | 0.55 | Tentative |
| P10909 | 2.82 | 2.88 | 0.10 | 4.85 | 0.60 | Confirmed |
| P04217 | 2.40 | 2.48 | -0.56 | 5.25 | 0.50 | Tentative |
| P02652 | 3.49 | 3.53 | -0.84 | 5.76 | 0.78 | Confirmed |
| P02649 | 3.76 | 3.87 | 0.43 | 5.59 | 0.81 | Confirmed |
| P08697 | 4.34 | 4.38 | -0.41 | 6.22 | 0.90 | Confirmed |
| P36955 | 4.73 | 4.82 | 1.00 | 6.67 | 0.92 | Confirmed |
| B9A064 | 4.55 | 4.63 | 0.87 | 6.66 | 0.91 | Confirmed |
| P05155 | 5.67 | 5.80 | 0.58 | 7.29 | 0.97 | Confirmed |
| P02750 | 3.72 | 3.85 | 0.27 | 5.62 | 0.81 | Confirmed |
| P01833 | 6.75 | 6.89 | 2.71 | 8.42 | 1.00 | Confirmed |
| Q9UK55 | 4.18 | 4.25 | 0.14 | 6.19 | 0.86 | Confirmed |
| P18428 | 2.38 | 2.39 | -0.22 | 5.06 | 0.45 | Tentative |
| P02654 | 4.24 | 4.37 | 0.02 | 6.20 | 0.88 | Confirmed |
| P06331 | 2.60 | 2.67 | -1.00 | 5.07 | 0.52 | Tentative |
| P35443 | 3.18 | 3.25 | 0.09 | 5.28 | 0.70 | Confirmed |
| P24592 | 3.61 | 3.72 | -0.49 | 6.05 | 0.77 | Confirmed |

**Abbreviations**: Imp- Importance measure computed over multiple iterations; Mean Imp- the mean of Imp; Median Imp- the median of Imp; Min Imp- the minimum of Imp; Max Imp- the maximum of Imp; Norm Hits- the number of hits normalized to number of importance source runs.

**Supplementary Table 5**: Proteomics studies for the identification of potential diagnostic biomarkers of IS using different biofluids

| **S. No** | **Study** | **Sample size** | **Differentiating condition** | **Specimen type** | **Sample collection time** | **Total proteins identified/ assayed** | **No. of sDEP** | **Proteomics approach** | **Remarks** |
| --- | --- | --- | --- | --- | --- | --- | --- | --- | --- |
| 1 | Malicek D, 2021(1) | IS=3, ICH=4, HC=2 | IS vs. ICH, IS vs. HC, ICH vs. HC, Total Stroke vs. HC | Plasma | Within 7 (1-15) days | 368 | 9 | SWATH-MS  (discovery) | 9 proteins significantly differentiated IS from ICH. |
| 2 | Wang W, 2021(2) | IS (CAS)=35, HC=18 | IS vs. HC | Urine | Not given | 1668 | 194 | DIA (discovery) | 194 proteins were sDEP between CAS and controls. |
| 3 | Zhang J, 2021(3) | IS=70, ICH=42, HC=65 | IS vs. ICH, ICH vs. HC | Metabolites | Within 5 days | 158 | 3 | UHPLC-MS (targeted) | 20-OH-LTB4 and arachidonic acid differentiated ICH from controls while 20-OH-LTB4 and 17,18-EpETE differentiated it from IS. |
| 4 | Lee J (a), 2020(4) | IS=20, HC=20 | IS vs. HC | Serum | Within 10 days | 399 | 13 | SWATH-MS (discovery) | 13 proteins were sDEP between IS and controls. |
| 5 | Lee J (b), 2020(5) | IS=18, HC=16 | IS vs. HC | Serum | Within 7 days | 203 | 110 | SWATH-MS (discovery) | Prothrombin, plasminogen, fibrinogen alpha chain and HRG were associated with coagulation cascade in IS cases. |
| 6 | Qin C, 2019(6) | IS (LVO)=40, HC=20 | IS vs. HC | Plasma | Within 7 days | Not given | 7 | iTRAQ (discovery) | 7 proteins were sDEP between IS and controls. |
| 7 | Cevik O, 2016(7) | IS=9, HC=9 | IS vs. HC | Platelets | Within 24 hours | 500 | 83 | UPLC-ESI-q-TOF-MS (discovery) | 83 proteins were sDEP between IS and controls. |
| 8 | Sharma R, 2015(8) | IS=20, HC=20 | IS vs. HC | Serum | Not given | 389 | 23 | iTRAQ (discovery) | 23 proteins were sDEP between IS and controls. |
| 9 | Dawson J, 2012(9) | IS/ TIA=65, HC=41 | IS/TIA vs. HC | Urine | Within 24 hours | Not given | 35 | CE-MS (discovery) | 35 proteins were sDEP between IS and controls. |
| 10 | Kodali P, 2012(10) | IS=10, ICH=9, Mimics=10 | IS vs. ICH, IS vs. Mimics, ICH vs. Mimics | Plasma | Within 12 hours | 38 | 8 | SEC-ICPMS (discovery) | 5 & 7 metals differentiated IS from mimics & ICH. |
| 11 | Lopez MZ, 2012(11) | IS=54, ICH=26, HC=31 | IS vs. ICH, IS vs. HC, ICH vs. HC | Plasma | Within 7 days | 9 | 4 | MRM (targeted) | ApoC-III, ApoC-I, ApoA-I, and ApoA-II differentiated IS, ICH, and control groups. |
| 12 | Brea D, 2011(12) | IS=11, HC=11 | IS vs. HC | Cells | Within 7 days | 406 | 4 | 2DE (discovery) | ERp29, CdC-42, eF2, and PRDX-1 were sDEP between IS and controls. |
| 13 | Allard L, 2004(13) | IS=11,  ICH=10, HC=21 | Total stroke vs. HC | Plasma | Within 72 hours | 7 peaks | 4 | SELDI | Apo C-1, Apo C-III, SAA, and AT-III fragment were sDEP between total stroke and controls. |

**Abbreviations**- IS: Ischemic Stroke, ICH: Intracerebral Hemorrhage, HC: Healthy Controls, LVO- Large Vessel Occlusion, CAS- Carotid Artery Stenosis, TIA- Transient Ischemic Attack, sDEP: significantly Differentially Expressed Proteins, UHPLC-MS: Ultra-High-Pressure Liquid Chromatography tandem Mass Spectrometry, MRM: Multiple Reaction Monitoring, SWATH-MS: Sequential Windowed Acquisition of all THeoretical fragment ion Mass Spectra, iTRAQ: isobaric Tags for Relative and Absolute Quantitation, UPLC-ESI-q-TOF-MS: Ultra Performance Liquid Chromatography- Electron Spray Ionization-q-Time-of-Flight- Mass Spectrometry, SEC-ICPMS: Size Exclusion Chromatography- Inductively Coupled Plasma Mass Spectrometry; DIA: Data Independent Acquisition, CE-MS: Capillary Electrophoresis-Mass Spectrometry, 2DE: 2 Dimensional Electrophoresis, SELDI: Surface-enhanced laser desorption/ Ionization, Apo: Apolipoprotein, 20-OH-LTB4: 20-hydroxy-leukotriene B4, 17,18-EpETE: 17, 18-epoxy-eicosatetraenoic acid, HRG: Histidine-rich glycoprotein, ERp29- Endoplasmatic Reticulum protein-29, PRDX-1: Peroxiredoxin-1, SAA: serum Amyloid A, AT-III fragment: Antithrombin-III fragment.

**Supplementary References**

1. Malicek D, Wittig I, Luger S, Foerch C. Proteomics-Based Approach to Identify Novel Blood Biomarker Candidates for Differentiating Intracerebral Hemorrhage From Ischemic Stroke-A Pilot Study. Front Neurol. 2021;12:713124.

2. Wang W, Wu J, Liu P, Tang X, Pang H, Xie T, et al. Urinary Proteomics Identifying Novel Biomarkers for the Diagnosis and Phenotyping of Carotid Artery Stenosis. Front Mol Biosci. 2021;8:714706.

3. Zhang J, Su X, Qi A, Liu L, Zhang L, Zhong Y, et al. Metabolomic profiling of fatty acid biomarkers for intracerebral hemorrhage stroke. Talanta. 2021 Jan 15;222:121679.

4. Lee J, Park A, Mun S, Kim HJ, Son H, Choi H, et al. Proteomics-Based Identification of Diagnostic Biomarkers Related to Risk Factors and Pathogenesis of Ischemic Stroke. Diagnostics (Basel). 2020 May 25;10(5):E340.

5. Lee J, Mun S, Park A, Kim D, Lee YJ, Kim HJ, et al. Proteomics Reveals Plasma Biomarkers for Ischemic Stroke Related to the Coagulation Cascade. J Mol Neurosci. 2020 Sep;70(9):1321–31.

6. Qin C, Zhao XL, Ma XT, Zhou LQ, Wu LJ, Shang K, et al. Proteomic profiling of plasma biomarkers in acute ischemic stroke due to large vessel occlusion. J Transl Med. 2019 Jul 1;17(1):214.

7. Cevik O, Baykal AT, Sener A. Platelets Proteomic Profiles of Acute Ischemic Stroke Patients. PLoS One. 2016;11(6):e0158287.

8. Sharma R, Gowda H, Chavan S, Advani J, Kelkar D, Kumar GSS, et al. Proteomic Signature of Endothelial Dysfunction Identified in the Serum of Acute Ischemic Stroke Patients by the iTRAQ-Based LC-MS Approach. J Proteome Res. 2015 Jun 5;14(6):2466–79.

9. Dawson J, Walters M, Delles C, Mischak H, Mullen W. Urinary proteomics to support diagnosis of stroke. PLoS One. 2012;7(5):e35879.

10. Kodali P, Chitta KR, Landero Figueroa JA, Caruso JA, Adeoye O. Detection of metals and metalloproteins in the plasma of stroke patients by mass spectrometry methods. Metallomics. 2012 Oct;4(10):1077–87.

11. Lopez MF, Sarracino DA, Prakash A, Athanas M, Krastins B, Rezai T, et al. Discrimination of ischemic and hemorrhagic strokes using a multiplexed, mass spectrometry-based assay for serum apolipoproteins coupled to multi-marker ROC algorithm. Proteomics Clin Appl. 2012 Apr;6(3–4):190–200.

12. Brea D, Rodríguez-González R, Sobrino T, Rodríguez-Yañez M, Blanco M, Castillo J. Proteomic analysis shows differential protein expression in endothelial progenitor cells between healthy subjects and ischemic stroke patients. Neurol Res. 2011 Dec;33(10):1057–63.

13. Allard L, Lescuyer P, Burgess J, Leung KY, Ward M, Walter N, et al. ApoC-I and ApoC-III as potential plasmatic markers to distinguish between ischemic and hemorrhagic stroke. Proteomics. 2004 Aug;4(8):2242–51.
